# Supplementary figures and images for: Reinforced education improves the quality of bowel preparation for colonoscopy: An updated meta-analysis of randomized controlled trials
Source: PLoS One. 2020 Apr 28;15(4):e0231888. doi: 10.1371/journal.pone.0231888 (PMC7188205; doi:10.1371/journal.pone.0231888)

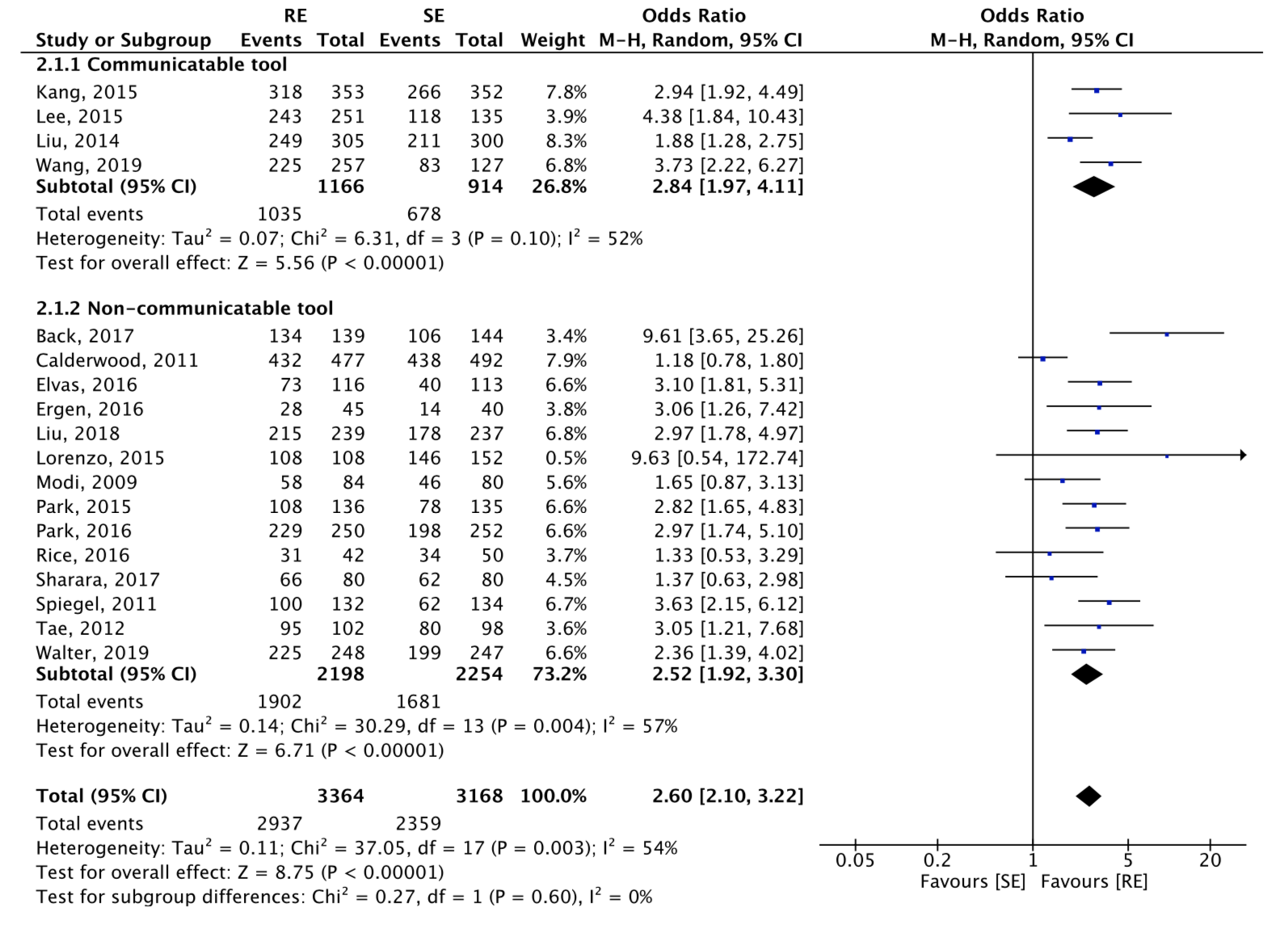

Supplement: S1 Fig — (TIFF) [file pone.0231888.s001.tiff]

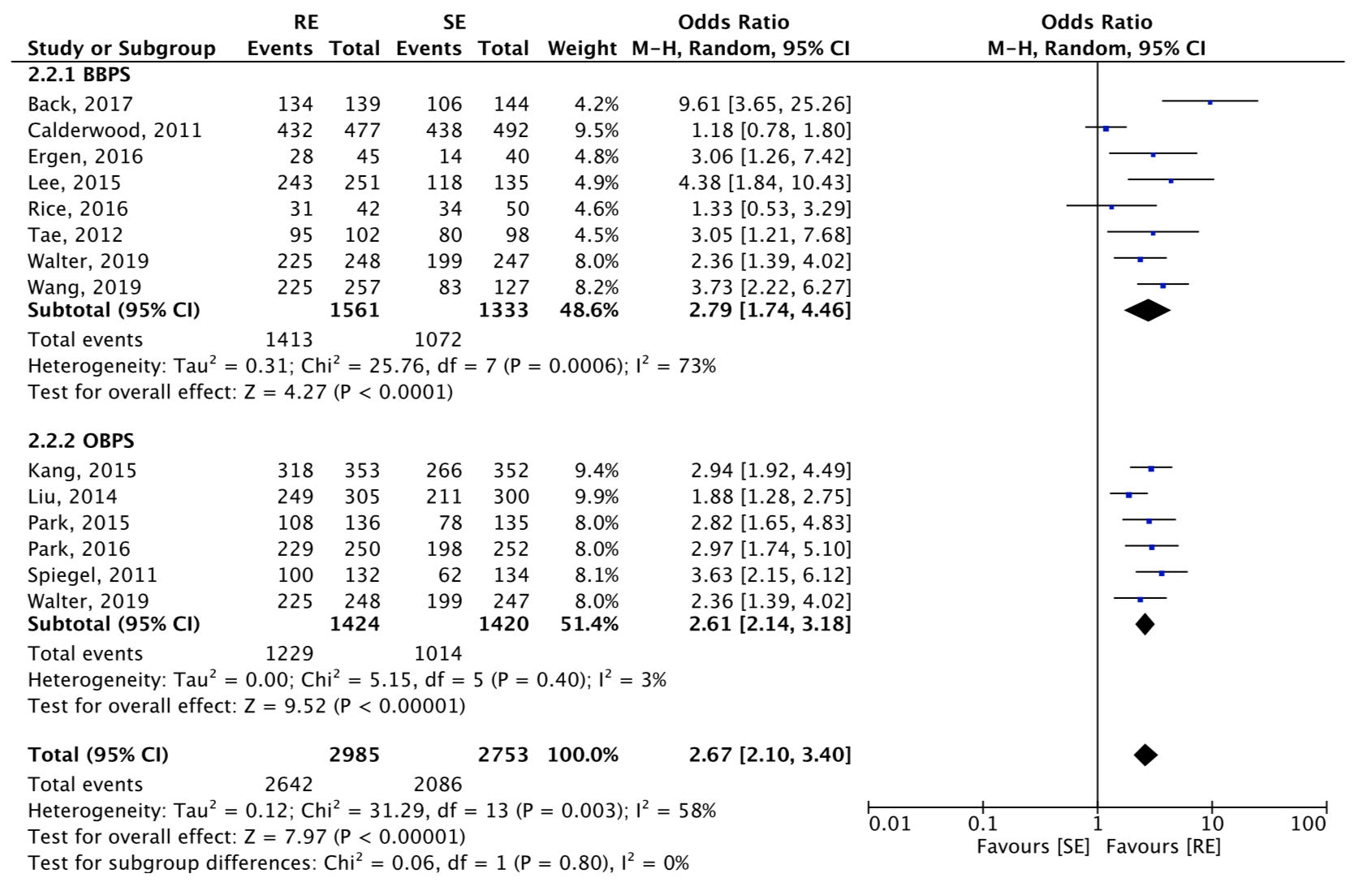

Supplement: S2 Fig — (TIFF) [file pone.0231888.s002.tiff]

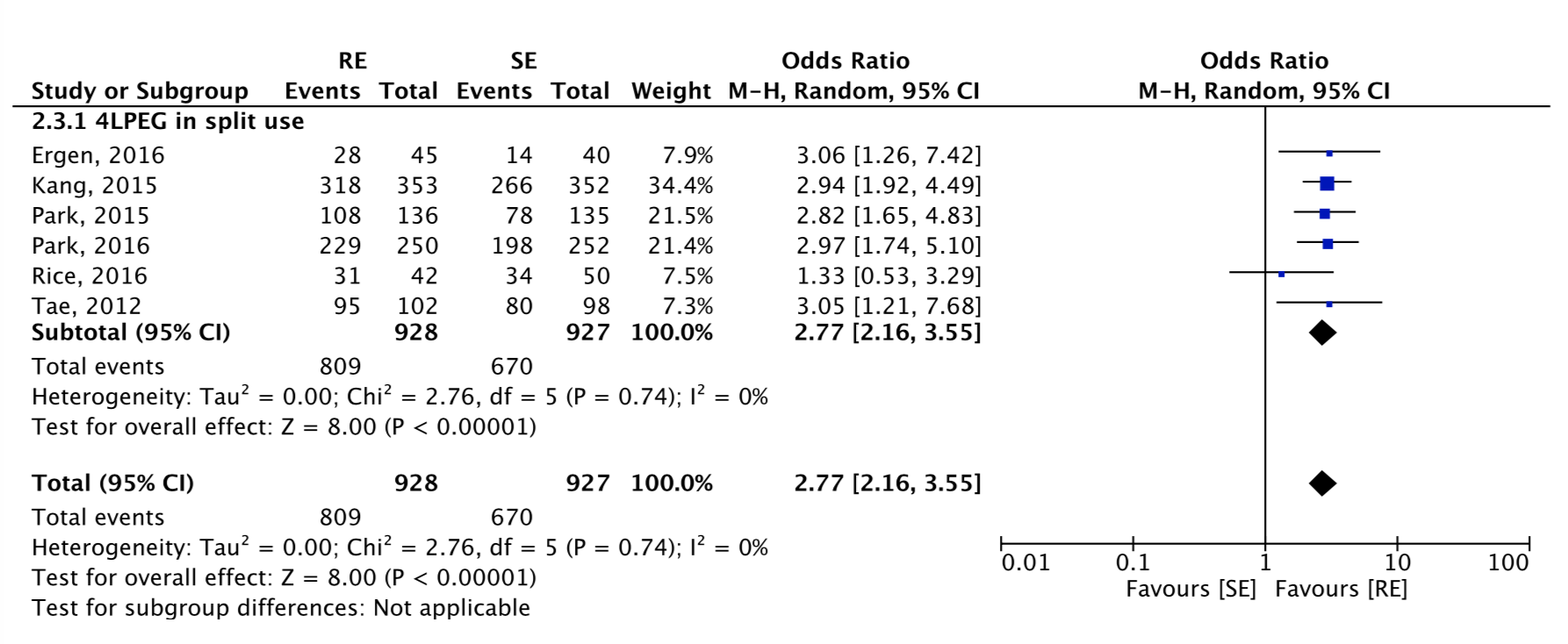

Supplement: S3 Fig — (TIFF) [file pone.0231888.s003.tiff]

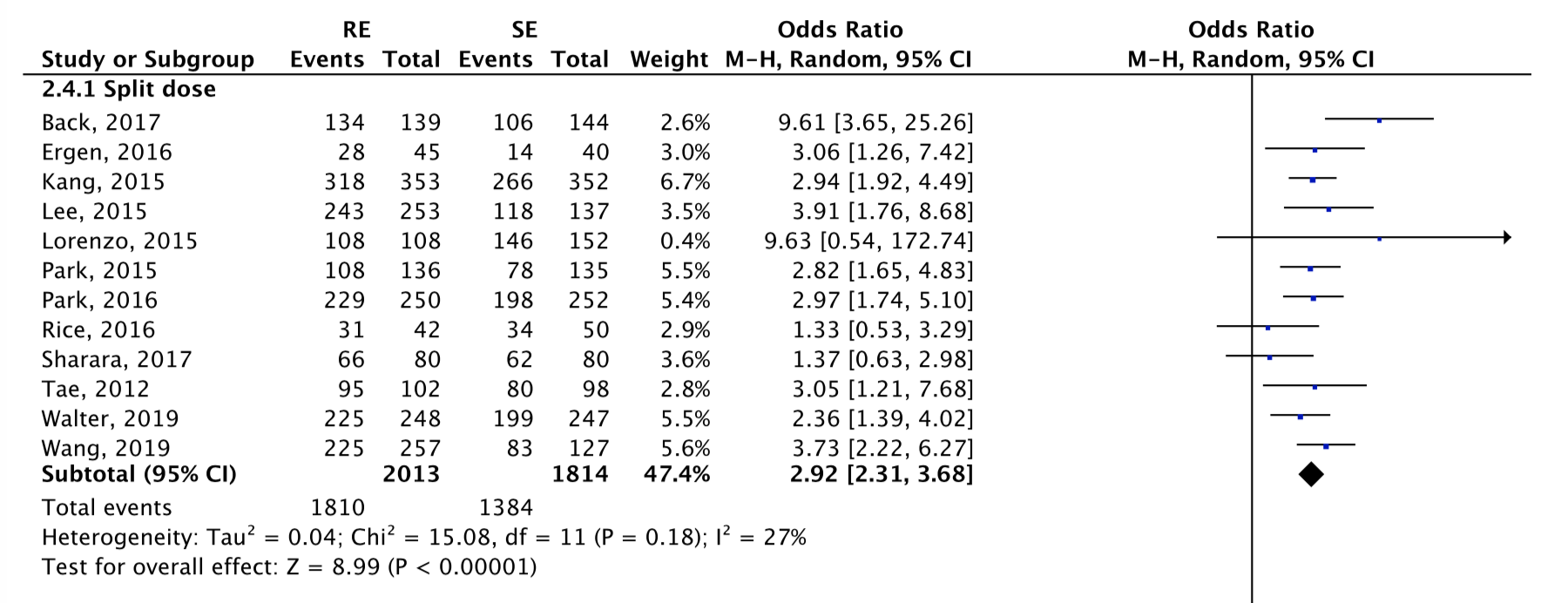

Supplement: S4 Fig — (TIFF) [file pone.0231888.s004.tiff]

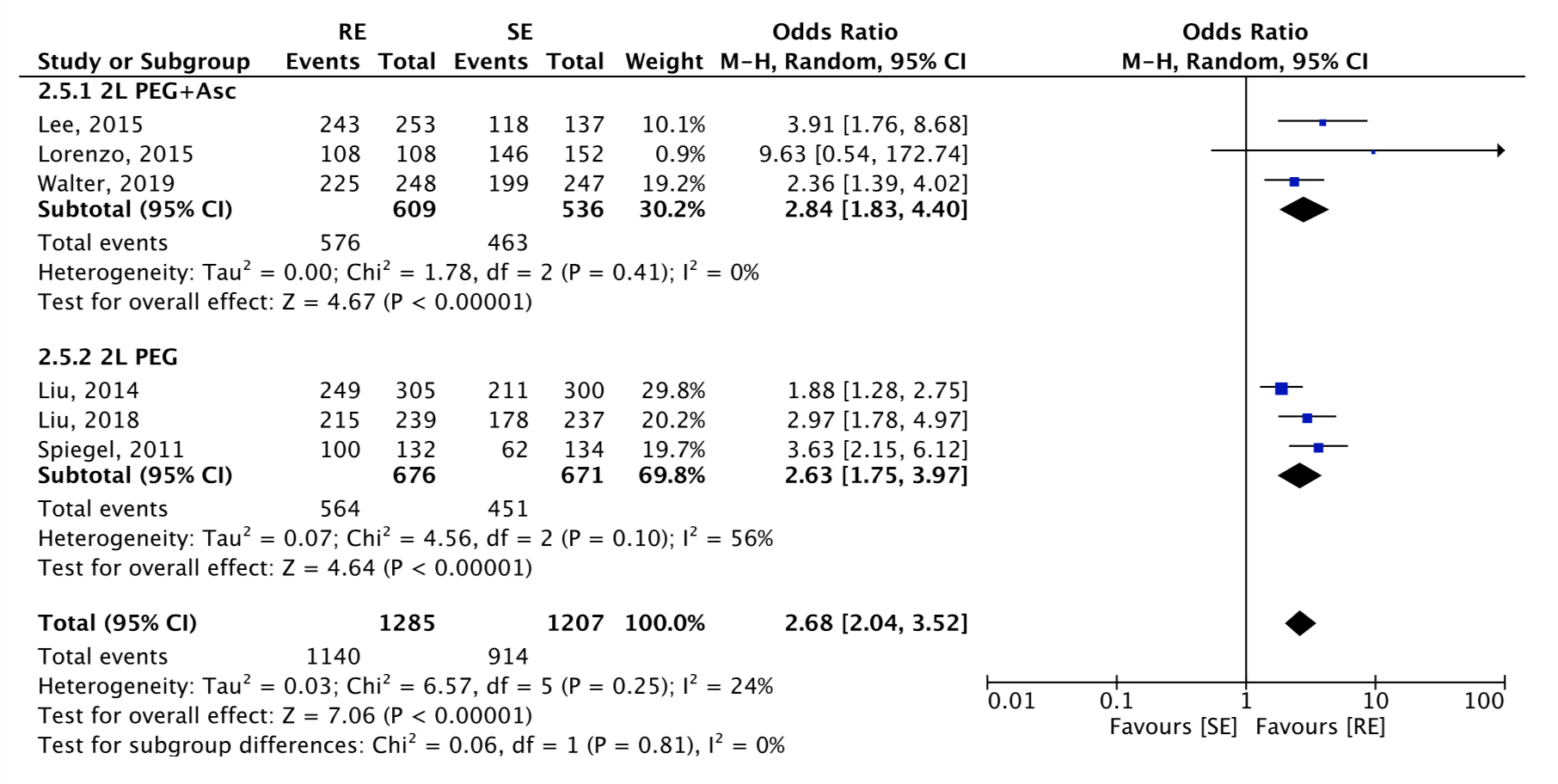

Supplement: S5 Fig — (TIFF) [file pone.0231888.s005.tiff]

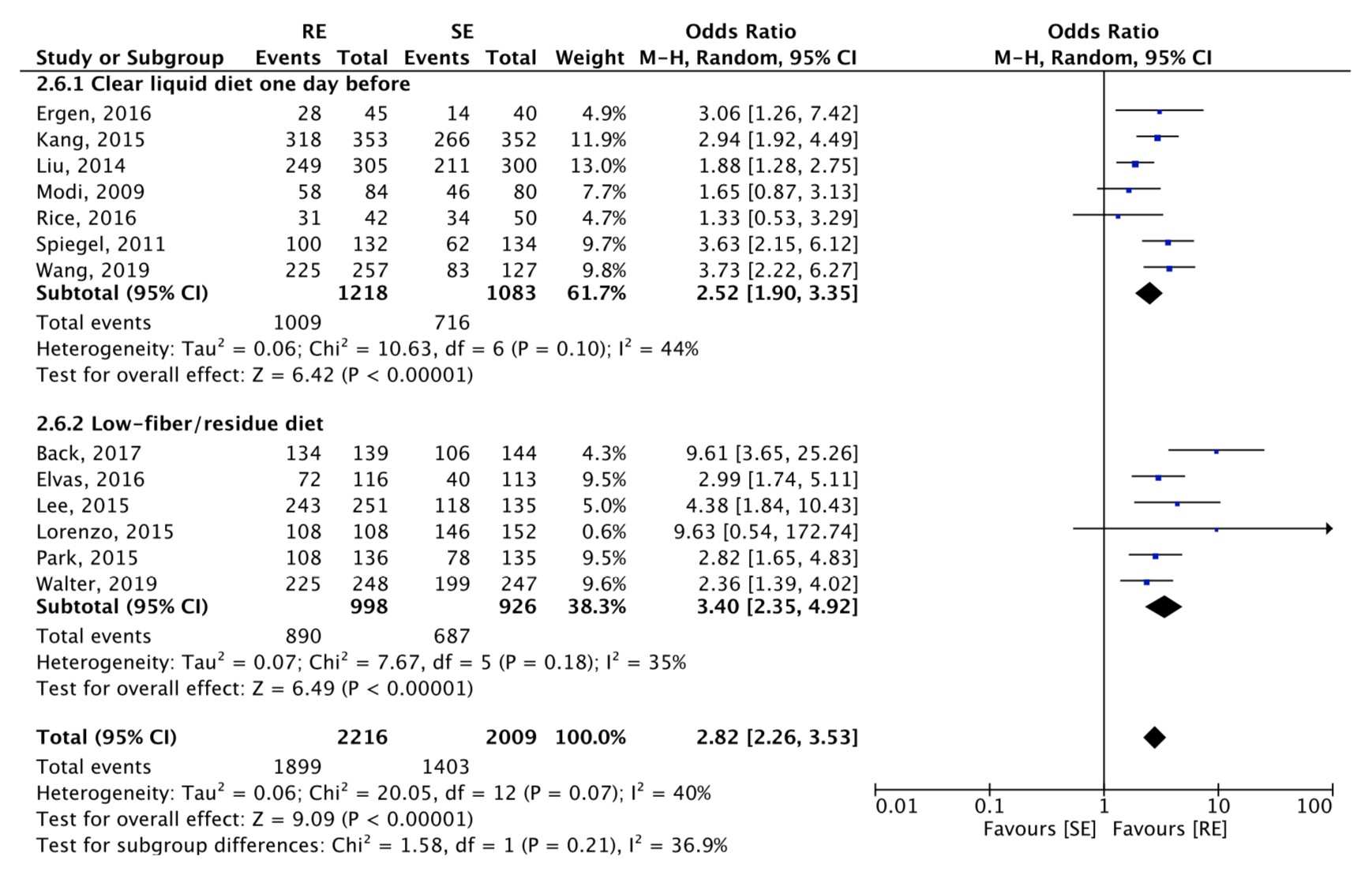

Supplement: S6 Fig — (TIFF) [file pone.0231888.s006.tiff]

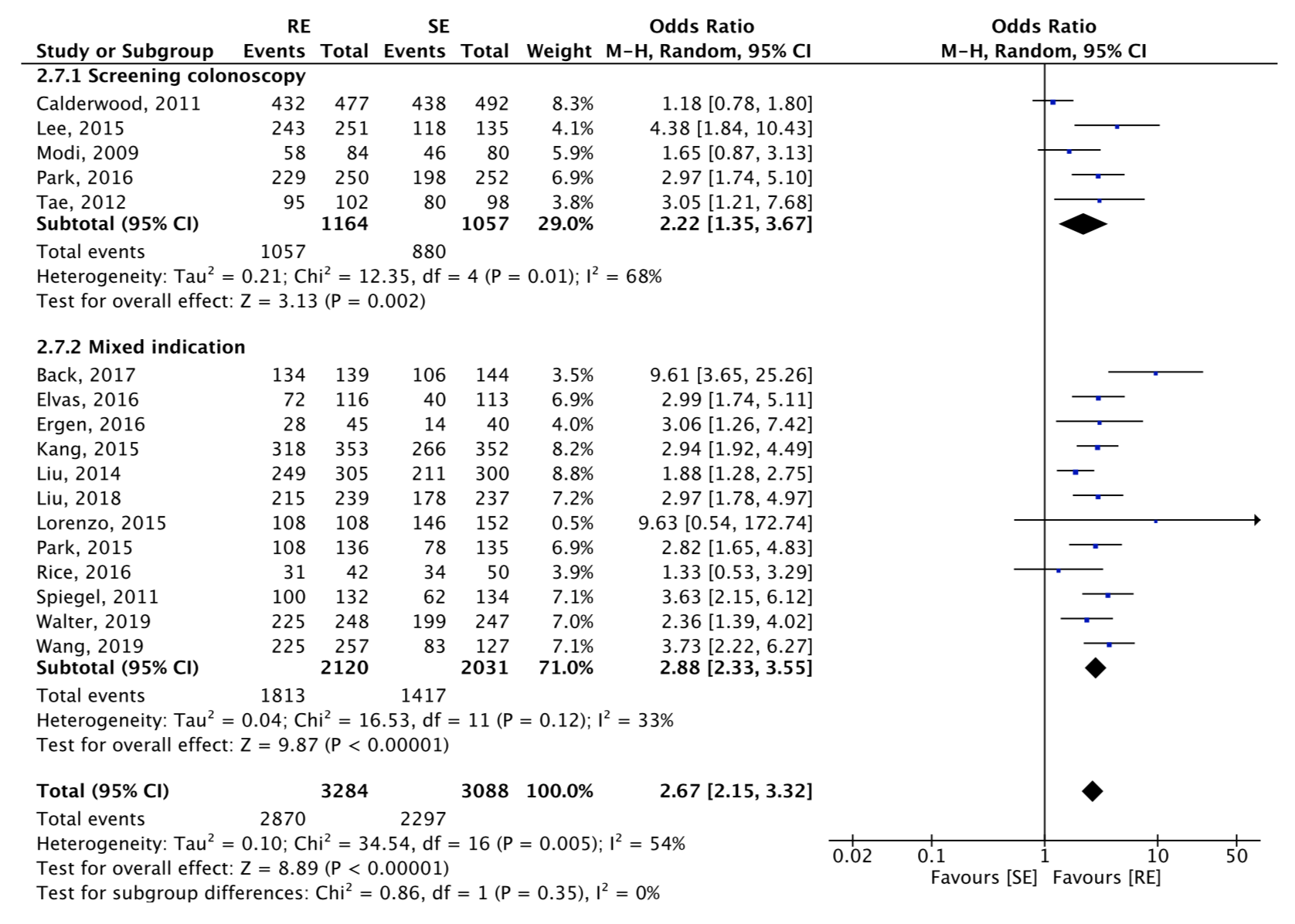

Supplement: S7 Fig — (TIFF) [file pone.0231888.s007.tiff]

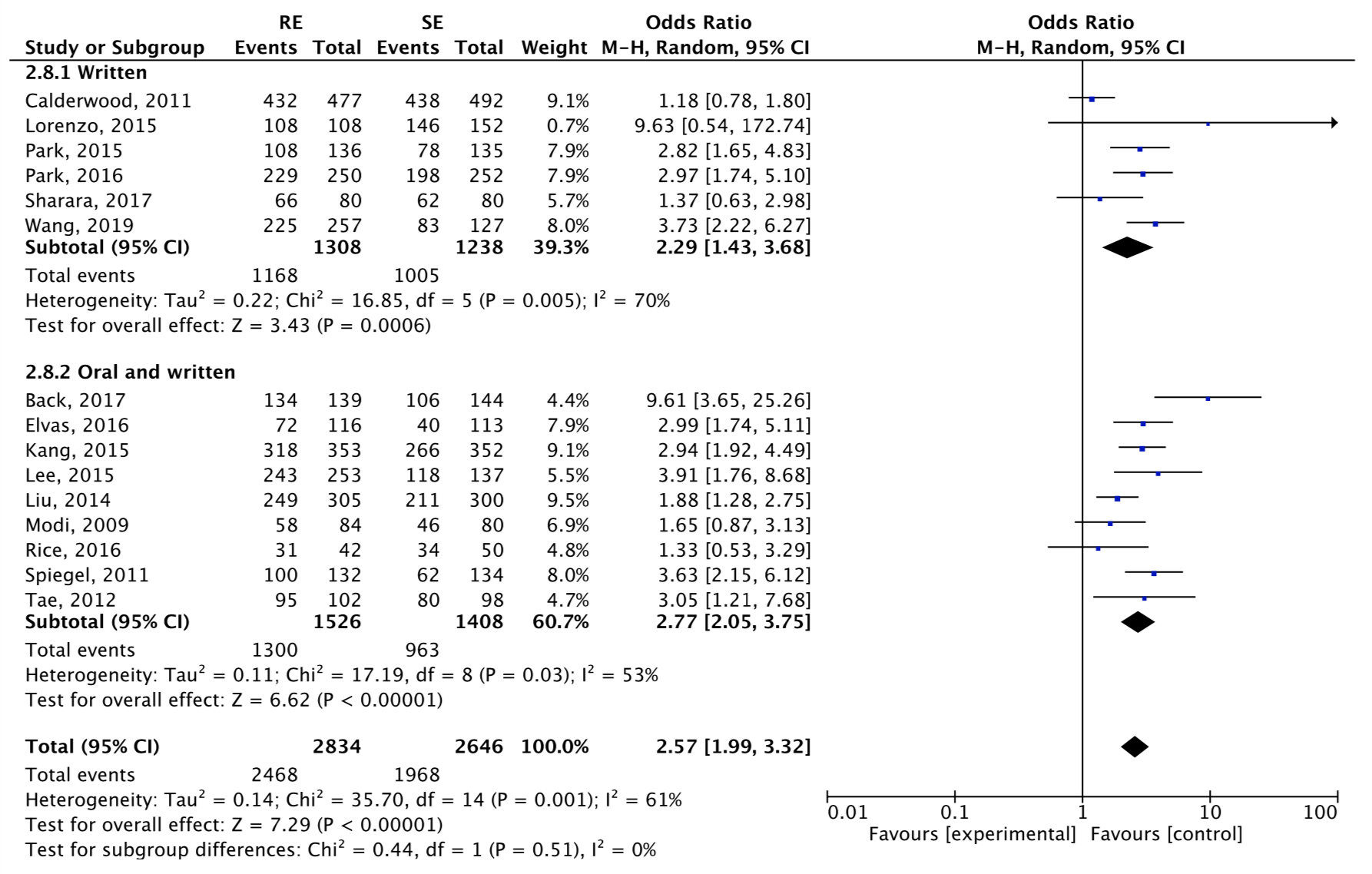

Supplement: S8 Fig — (TIFF) [file pone.0231888.s008.tiff]

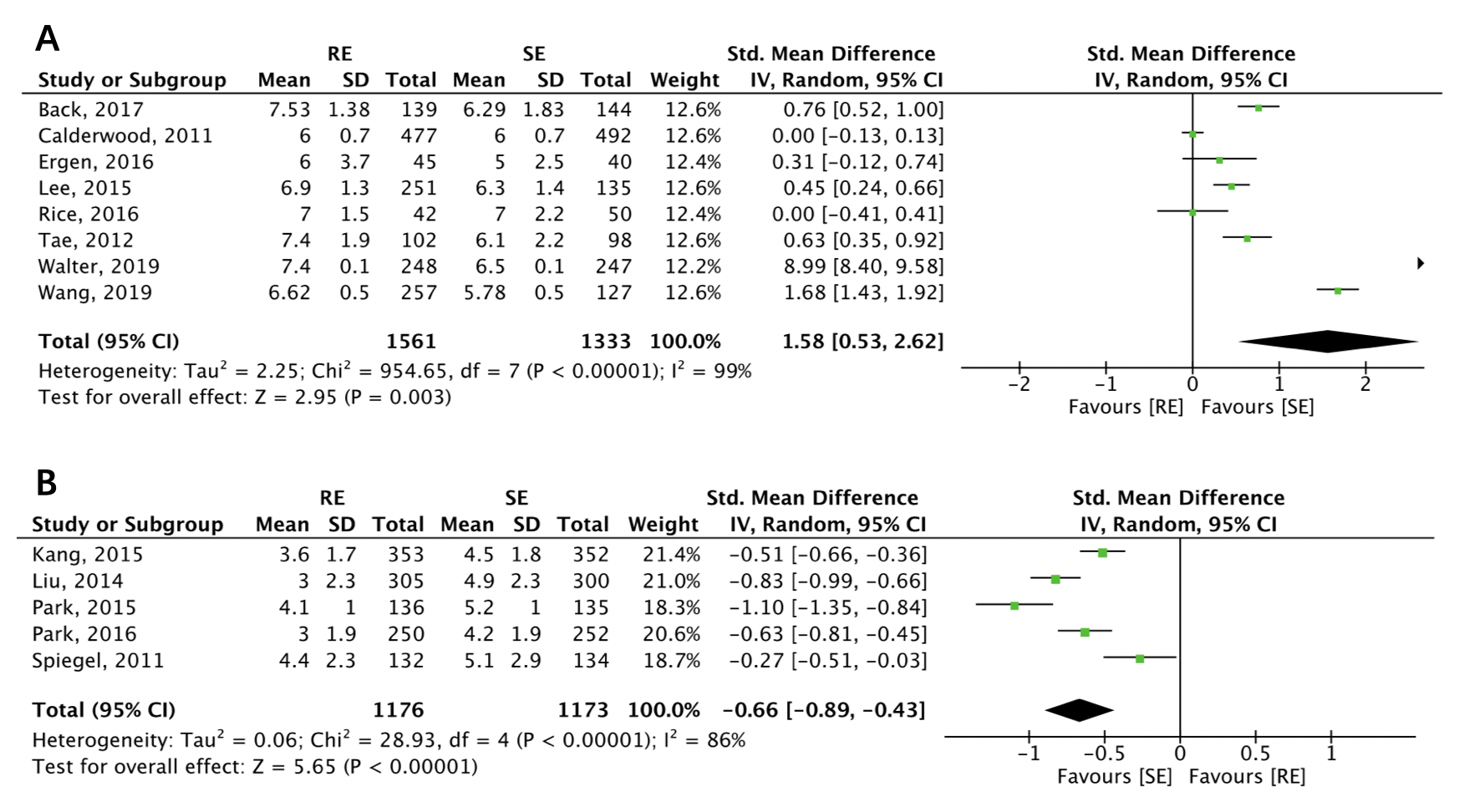

Supplement: S9 Fig — (TIFF) [file pone.0231888.s009.tiff]

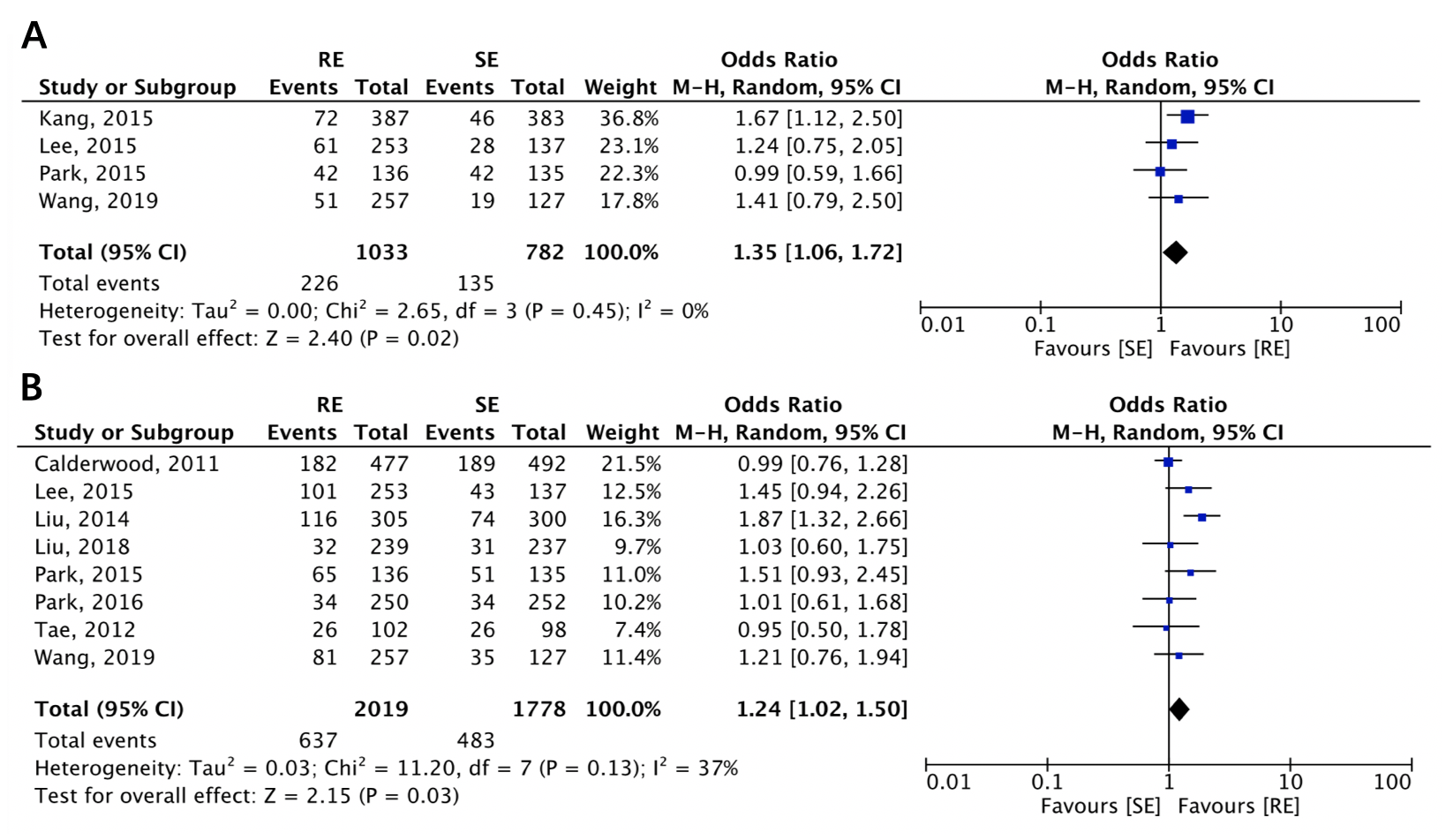

Supplement: S10 Fig — (TIFF) [file pone.0231888.s010.tiff]

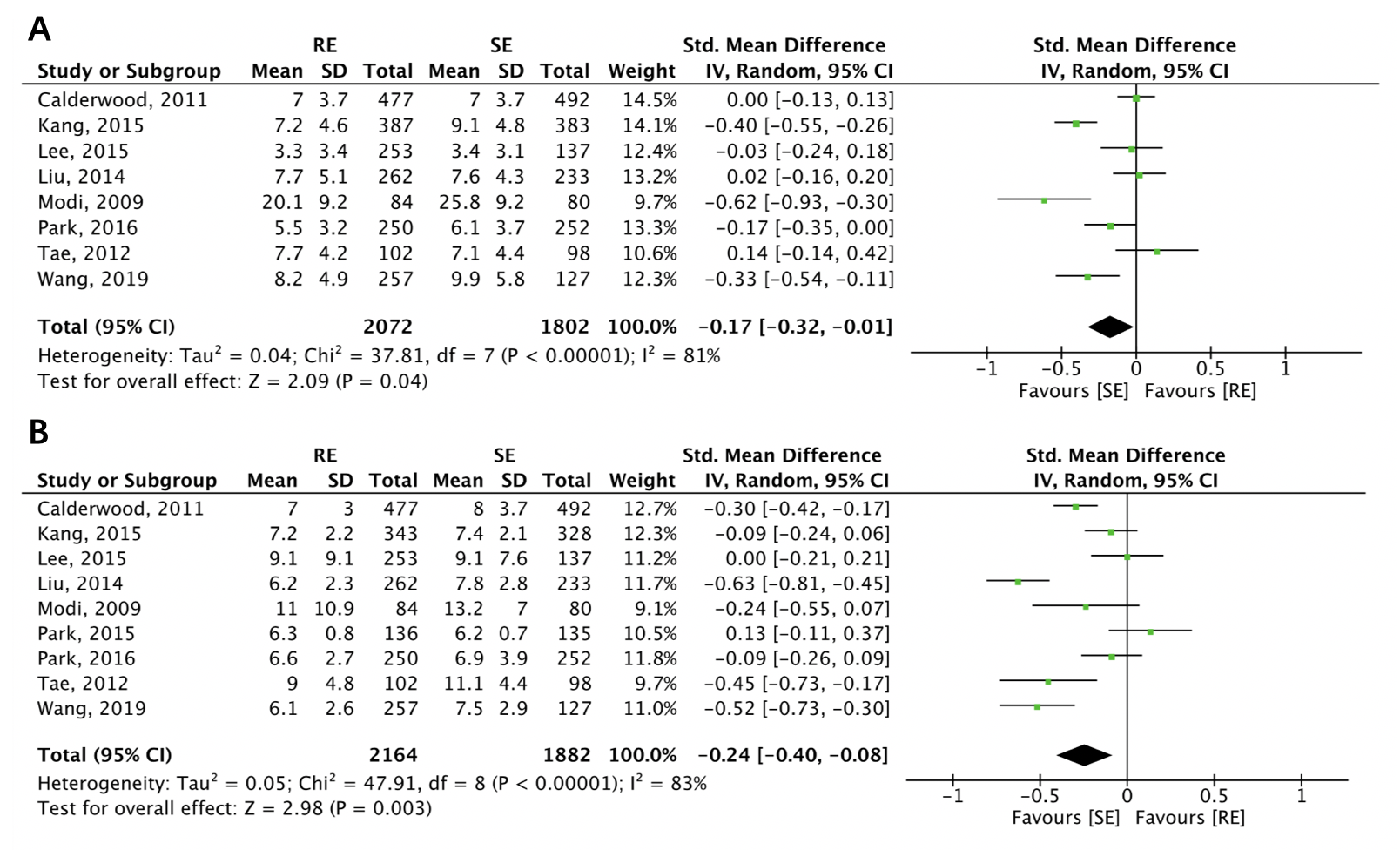

Supplement: S11 Fig — (TIFF) [file pone.0231888.s011.tiff]

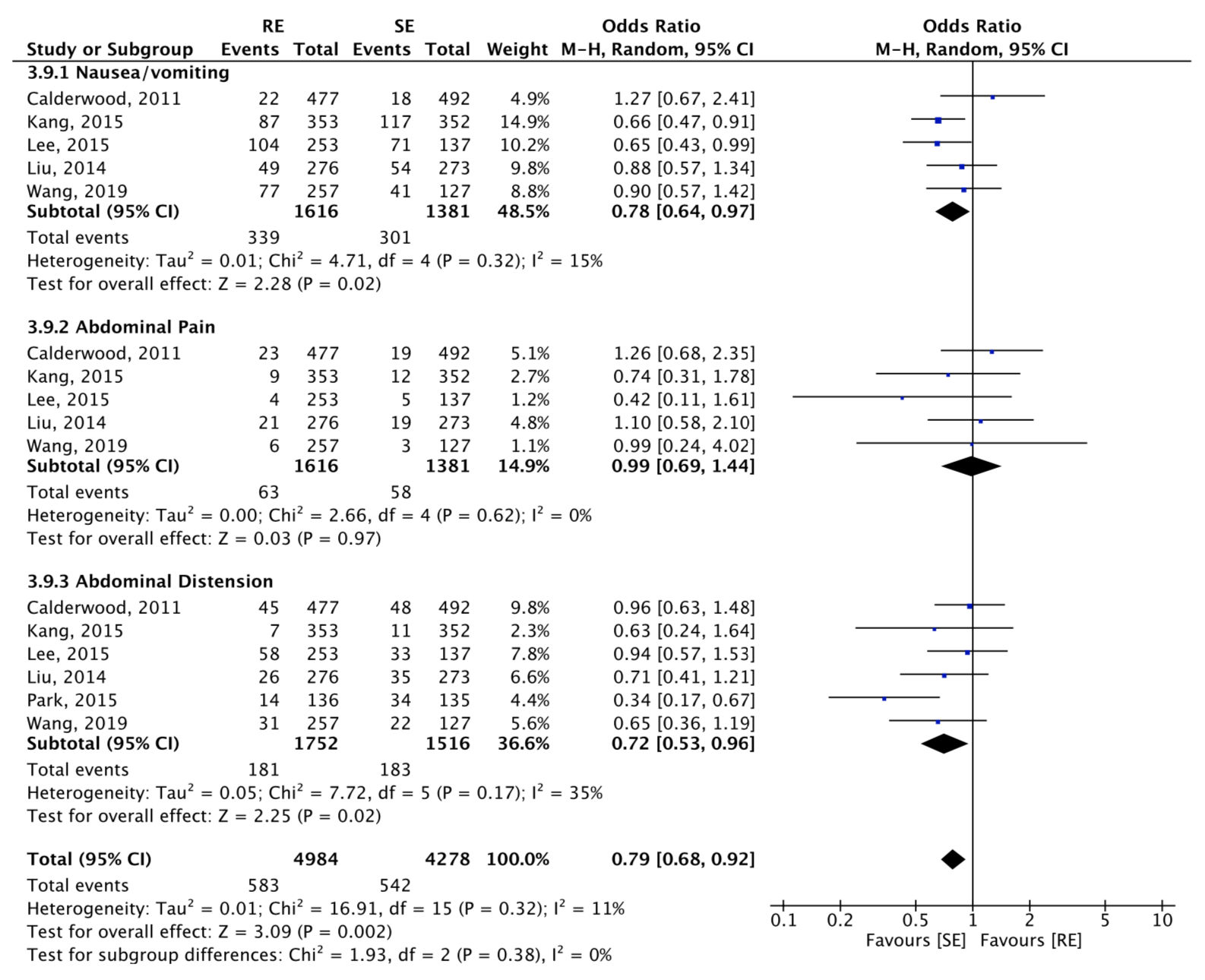

Supplement: S12 Fig — (TIFF) [file pone.0231888.s012.tiff]

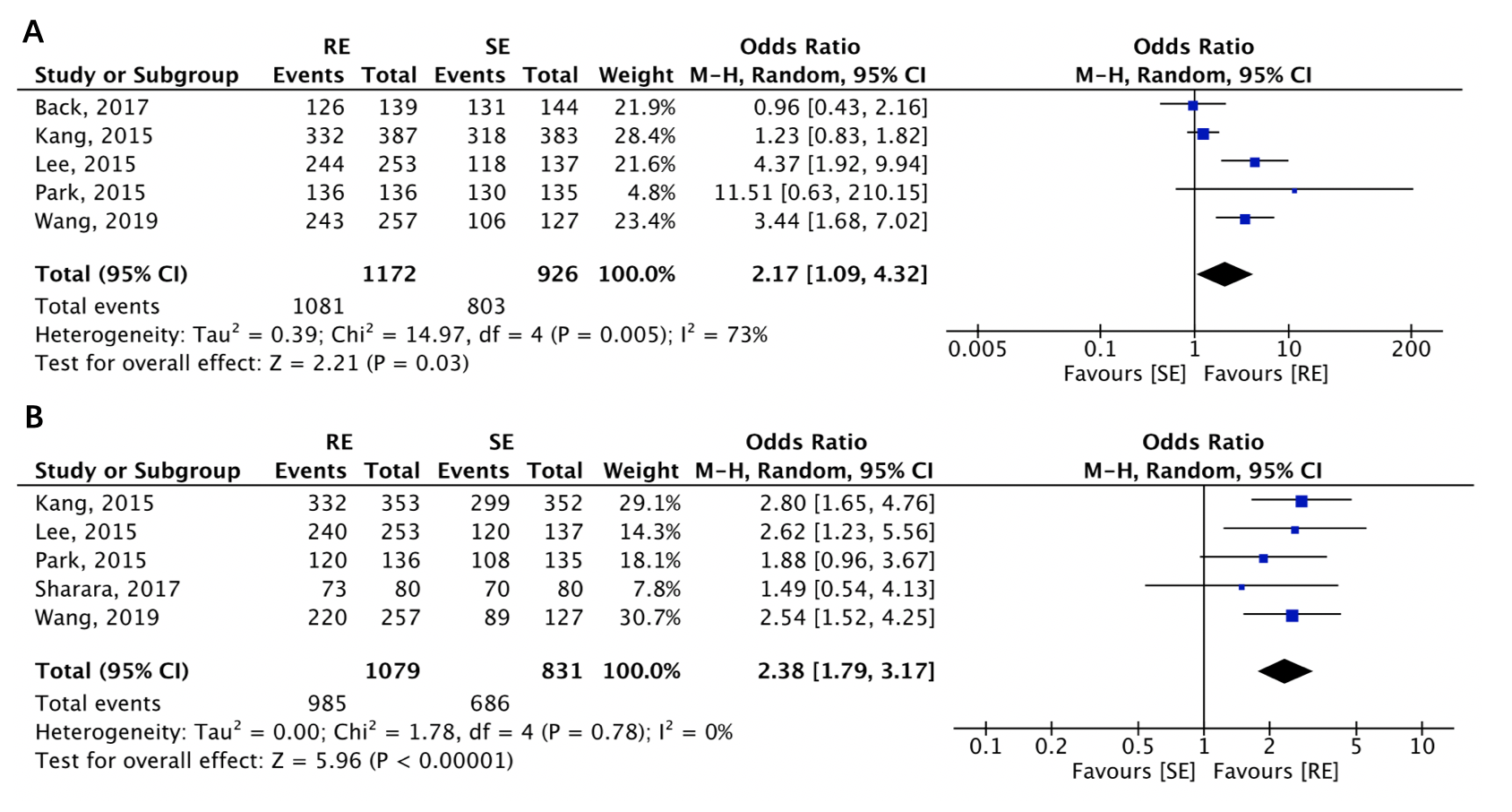

Supplement: S13 Fig — (TIFF) [file pone.0231888.s013.tiff]

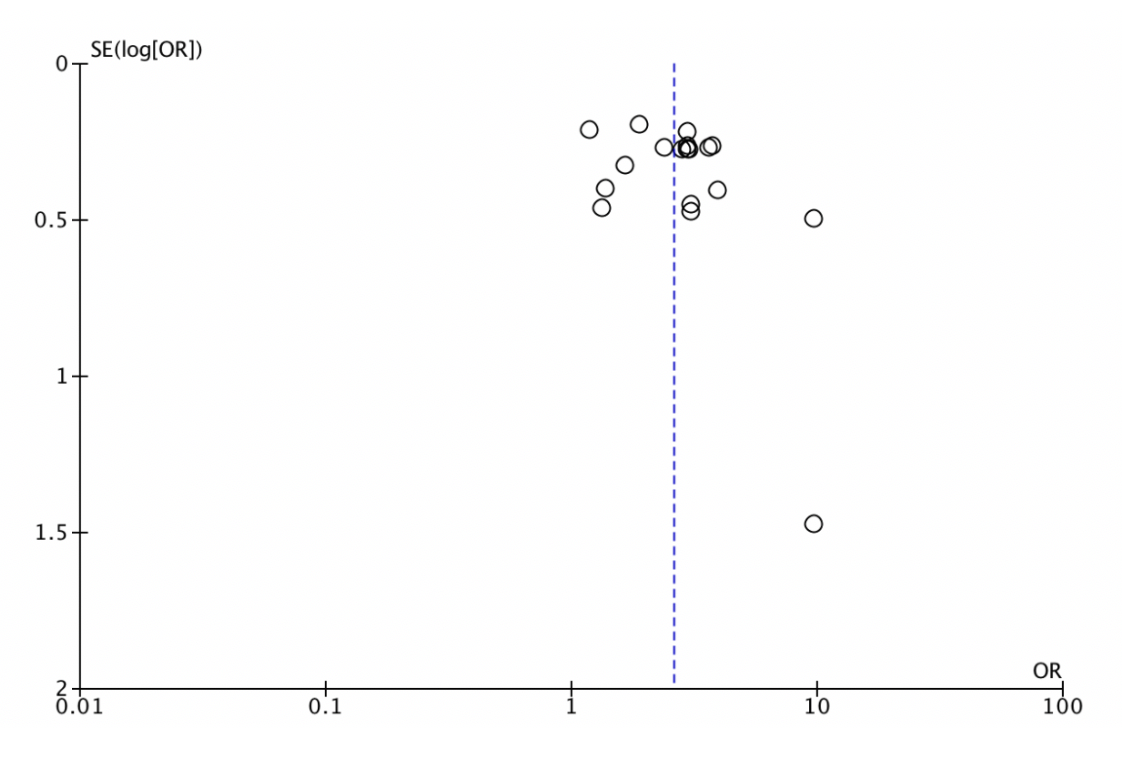

Supplement: S14 Fig — (TIFF) [file pone.0231888.s014.tiff]

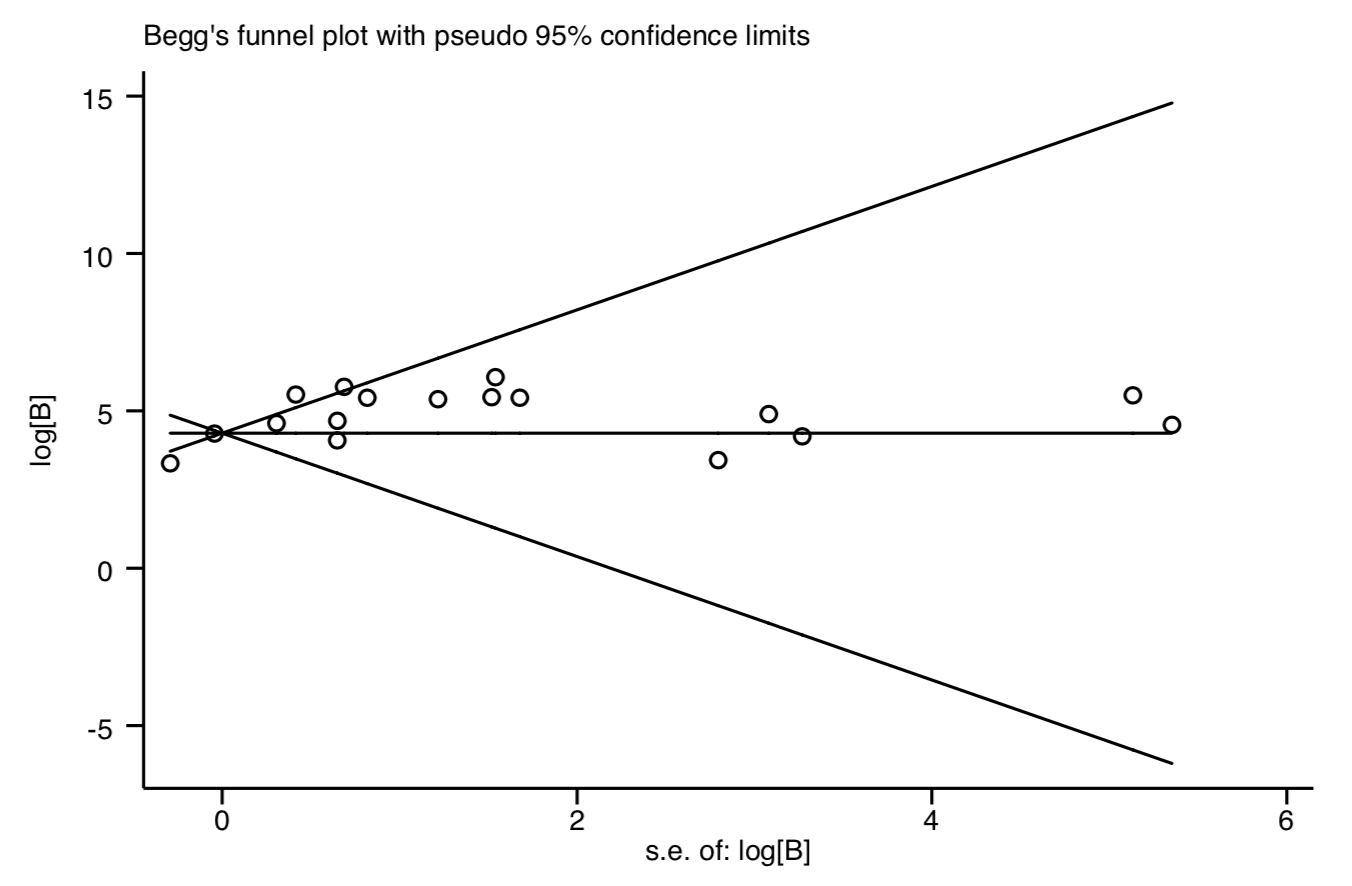

Supplement: S15 Fig — (TIFF) [file pone.0231888.s015.tiff]

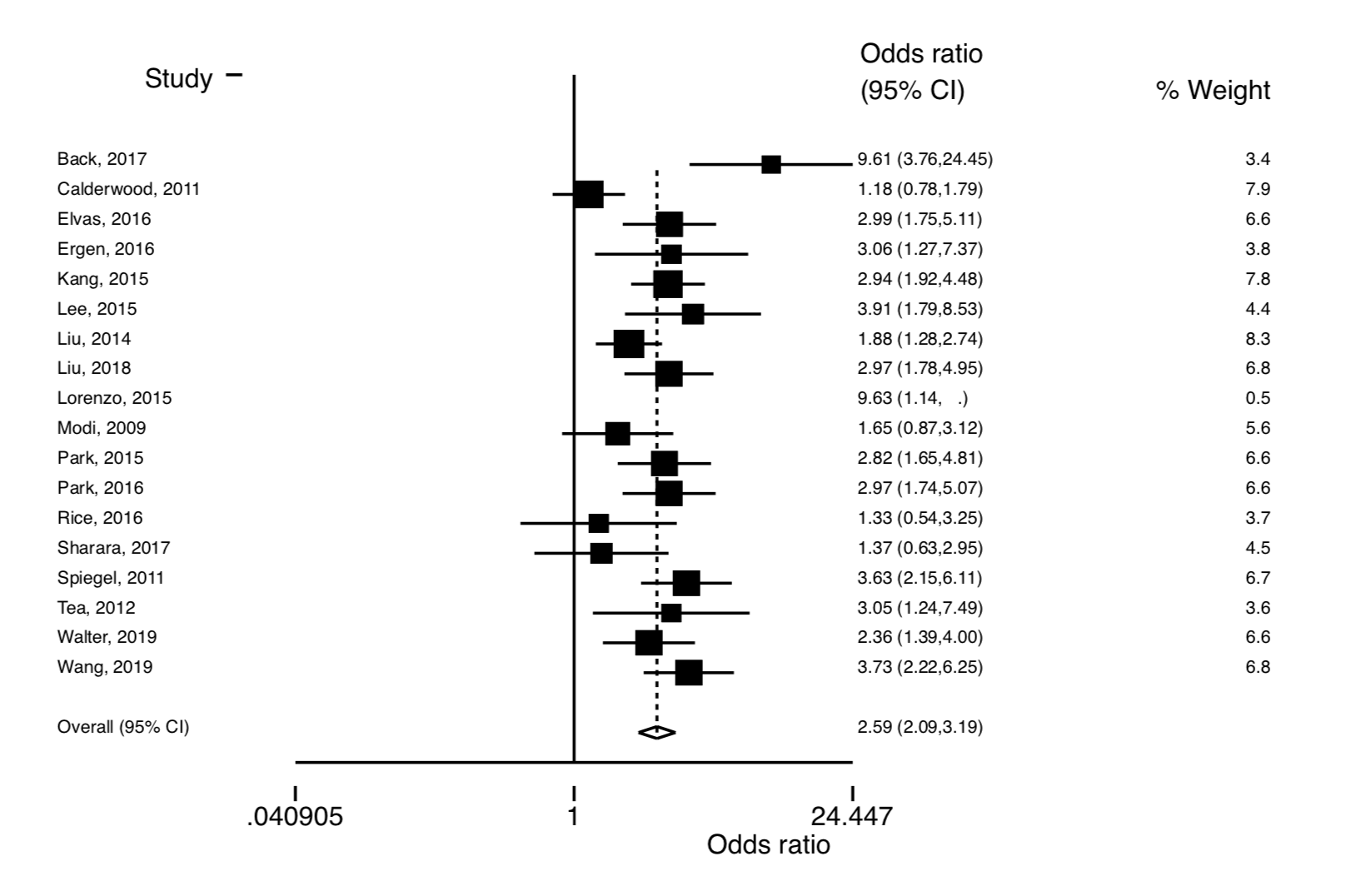

Supplement: S16 Fig — (TIFF) [file pone.0231888.s016.tiff]
