# Supplementary material for: Reinforced education improves the quality of bowel preparation for colonoscopy: An updated meta-analysis of randomized controlled trials
Source: PLoS One. 2020 Apr 28;15(4):e0231888. doi: 10.1371/journal.pone.0231888 (PMC7188205; doi:10.1371/journal.pone.0231888)
Supplement: S1 Table — (DOCX) [file pone.0231888.s017.docx]

Supplementary table 1. Details of each included study

| Study | BP scale | BP score, mean (SD) | Purgative | Administration method | Diet | Insertion time, mean (SD) | Withdrawal time, mean (SD) | ADR | PDR | ＞80% of purgative ingested | Diet compliance |
| --- | --- | --- | --- | --- | --- | --- | --- | --- | --- | --- | --- |
| Back, 2018 | BBPS | 7.53(1.38) vs. 6.29(1.83) | 4L  PEG or 2L PEG + Asc or SPMC | Split | LFD 3 days before | NR | NR | NR | NR | 126/139 vs. 131/144 | NR |
| Calderwood, 2011 | BBPS | 6.0(0.7) vs. 6.0(0.7) | 4L PEG or 4L PEG + bisacodyl | NR | NR | 7.0(3.7) vs. 7.0(3.7) | 7.0(3.0) vs. 8.0(3.7) | NR | 182/477 vs. 189/492 | NR | NR |
| Ergen, 2016 | BBPS | 6.0(3.7) vs. 5.0(2.5) | 4L PEG | Split | CLD 1 day before | NR | NR | NR | NR | NR | NR |
| Elvas, 2016 | Aronchick scale | NR | 4L PEG | Single | LFD 1 day before | NR | NR | NR | NR | NR | NR |
| Kang, 2016 | OBPS | 3.6(1.7) vs. 4.5(1.8) | 4L PEG | Split | CLD 1 day before | 7.2(4.6) vs. 9.1(4.8) | 7.2(2.2) vs. 7.4(2.1) | 72/387 vs. 46/383 | NR | 332/387 vs. 318/383 | 332/387 vs. 299/383 |
| Lee, 2015 | BBPS | 6.9(1.3) vs. 6.3(1.4) | 2L PEG + Asc | Split | LRD 2 days before | 3.3(3.4) vs. 3.4(3.1) | 9.1(9.1) vs. 9.1(7.6) | 61/253 vs. 28/137 | 101/253 vs. 43/137 | 244/253 vs. 118/137 | 240/253 vs. 120/137 |
| Liu, 2014 | OBPS | 3.0(2.3) vs. 4.9(3.2) | 2L PEG or 1.5L NaP | Single | CLD 1 day before | 7.7(5.1) vs. 7.6(4.3) | 6.2(2.3) vs. 7.8(2.8) | NR | 116/305 vs. 74/300 | NR | NR |
| Liu, 2018 | OBPS | 3.05(1.30) vs. 4.18(1.40) | 2L PEG | Single | CLD 1 day before | 5.1(4.8) vs. 6.0(4.2) | 6.8(2.5) vs. 7.0(3.2) | NR | 32/239 vs. 31/237 | NR | NR |
| Lorenzo, 2015 | HCS | 16.5(3.1) vs. 17.1(3.2) | 2L PEG + Asc | Split | LFD 1 day before | NR | NR | NR | 23/108 vs. 31/152 | NR | NR |
| Modi, 2009 | UPAS | NR | 4L PEG+ bisacodyl | NR | CLD 1 day before | 20.1(9.1) vs. 25.8(9.2) | 11.0(10.9) vs. 13.2(7.0) | NR | NR | NR | NR |
| Park, 2015 | OBPS | 4.1(1.0) vs. 5.2(1.0) | 4L PEG | Split | LFD 3 days before | NR | 6.3(0.8) vs. 6.2(0.7) | 42/136 vs. 42/135 | 65/136 vs. 51/135 | 136/136 vs. 130/135 | 120/136 vs. 108/135 |
| Park, 2016 | OBPS | 3.0(1.9) vs. 4.2(1.9) | 4L PEG | Split | Regular meal 1 day before | 5.5(3.2) vs. 6.1(3.7) | 6.6(2.7) vs. 6.9(3.9) | NR | 34/250 vs. 34/252 | NR | NR |
| Rice, 2016 | BBPS | 7.0(1.5) vs. 7.0(2.2) | 4L PEG | Split | CLD 1 day before | NR | NR | NR | NR | NR | NR |
| Sharara, 2017 | Aronchick scale | NR | SPMC | Split | LFD 3 days before and CLD 1 day before | NR | NR | NR | NR | NR | 72/80 vs. 66/80 |
| Spiegel, 2011 | OBPS | 4.4(2.3) vs. 5.1(2.9) | 2L PEG or NaP or magnesium citrate | Single | CLD 1 day before | NR | NR | NR | NR | NR | NR |
| Tae, 2012 | BBPS | 7.4(1.9) vs. 6.1(2.2) | 4L PEG | Split | NR | 7.7(4.2) vs. 7.1(4.4) | 9.0(4.8) vs. 11.1(4.4) | NR | 26/102 vs.26/98 | NR | NR |
| Walter, 2019 | BBPS | 7.4(0.1) vs. 6.5(0.1) | 2L PEG + Asc | Split | LFD 3 days before | NR | 7.8(0.1) vs. 7.7(0.1) | NR | NR | NR | NR |
| Wang, 2019 | BBPS | 6.62(0.50) vs. 5.78(0.50) | 3L PEG | Split | CLD 1 day before | 8.2(4.9) vs. 9.9(5.8) | 6.1(2.6) vs. 7.5(2.9) | 51/257 vs. 19/127 | 81/257 vs. 35/127 | 243/257 vs. 106/127 | 220/257 vs. 89/127 |

RE, reinforced education; SE, standard education; BP, bowel preparation; SD, standard deviation; ADR, adenoma detection rate; PDR, polyp detection rate; NR, not reported; BBPS, Boston Bowel Preparation Scale; OBPS, Ottawa Bowel Preparation Scale; UBAS, Universal Bowel Assessment Scale; HCS, Harefield Cleansing Scale; PEG, polyethylene glycol; SPMC, sodium picosulfate with magnesium citrate; Asc, ascorbic acid; LFD, low-fiber diet; CLD, clear liquid diet; LRD, low-residue diet
