# Supplementary material for: Reinforced education improves the quality of bowel preparation for colonoscopy: An updated meta-analysis of randomized controlled trials
Source: PLoS One. 2020 Apr 28;15(4):e0231888. doi: 10.1371/journal.pone.0231888 (PMC7188205; doi:10.1371/journal.pone.0231888)
Supplement: S2 Table — (DOC) [file pone.0231888.s018.doc]

Supplementary table 2. Details of quality assessment using modified Jadad score

| Study | Randomization | Concealment of allocation | Double blinding | Description of withdrawals and dropouts | Total Withdrawals and dropouts |
| --- | --- | --- | --- | --- | --- |
| Back, 2017 | 2 | 2 | 1 | 1 | 6 |
| Calderwood, 2011 | 2 | 2 | 1 | 1 | 6 |
| Ergen, 2016 | 2 | 2 | 1 | 1 | 6 |
| Elvas, 2016 | 2 | 2 | 1 | 1 | 6 |
| Kang, 2015 | 2 | 2 | 1 | 1 | 6 |
| Lee, 2015 | 2 | 2 | 1 | 1 | 6 |
| Liu, 2014 | 2 | 2 | 1 | 1 | 6 |
| Liu, 2018 | 2 | 2 | 1 | 0 | 5 |
| Lorenzo, 2015 | 1 | 1 | 1 | 1 | 4 |
| Modi, 2009 | 1 | 1 | 1 | 0 | 3 |
| Park, 2015 | 2 | 2 | 1 | 1 | 6 |
| Park, 2016 | 2 | 2 | 1 | 1 | 6 |
| Rice, 2016 | 2 | 2 | 1 | 1 | 6 |
| Sharara, 2017 | 2 | 2 | 1 | 1 | 6 |
| Spiegel, 2011 | 2 | 2 | 1 | 1 | 6 |
| Tae, 2012 | 2 | 2 | 1 | 1 | 6 |
| Walter, 2019 | 2 | 2 | 1 | 1 | 6 |
| Wang, 2019 | 2 | 2 | 1 | 1 | 6 |
