# Supplementary material for: Reinforced education improves the quality of bowel preparation for colonoscopy: An updated meta-analysis of randomized controlled trials
Source: PLoS One. 2020 Apr 28;15(4):e0231888. doi: 10.1371/journal.pone.0231888 (PMC7188205; doi:10.1371/journal.pone.0231888)
Supplement: S3 Table — (DOCX) [file pone.0231888.s019.docx]

Supplementary table 3. Bowel preparation quality in patients receiving split-dose with any laxatives.

|  | Studies | Adequate rate of bowel preparation | | | | BBPS score | | | |
| --- | --- | --- | --- | --- | --- | --- | --- | --- | --- |
|  |  | RE | SE | 95%CI | P value | Mean score | SMD | 95%CI | P value |
| Any laxatives | 12 | 90.0% | 76.3% | 2.31-3.68 | <0.001 | 6.71 vs. 6.23 | 1.81 | 0.50-3.12 | 0.007 |
| 3L or 4L PEG | 8 | 88.2% | 71.7% | 2.40-4.06 | <0.001 | 6.95 vs. 6.07 | 0.69 | 0.13-2.09 | 0.02 |
| 2L PEG+Asc | 4 | 94.9% | 83.7% | 2.08-8.77 | <0.001 | 7.23 vs. 6.39 | 3.38 | 0.15-6.61 | 0.04 |
| SPMC | 2 | 91.3% | 75.0% | 0.20 |  |  |  |  |  |
