# Supplementary material for: Reinforced education improves the quality of bowel preparation for colonoscopy: An updated meta-analysis of randomized controlled trials
Source: PLoS One. 2020 Apr 28;15(4):e0231888. doi: 10.1371/journal.pone.0231888 (PMC7188205; doi:10.1371/journal.pone.0231888)
Supplement: Attachment — Submitted filename: response letter.docx [file pone.0231888.s021.docx]

Dear editor,

We thank the editor and reviewers for reviewing our Manuscript ID PONE-D-19-27355 entitled “Reinforced education improves the quality of bowel preparation for colonoscopy: an updated meta-analysis of randomized controlled trials”. We have revised the manuscript according to the constructive suggestions. A point-by-point response is included below.

In response to the reviewers' comments, additional information has been added to the revised manuscript. These are marked by red highlights in the version with tracked changes. The version with all changes accepted (clean version) are also provided. Deleted text no longer appears in the final clean version.

**Editor**

1, A rigorous statistical analysis is clue in this type of manuscripts. I agree with the statistician that it needs improvement and better describe in more detail. Please pay a very careful attention to the statistician comments. As mentioned by the statistician, currently the manuscript do not meet the *Plos One* criteria (see publication criteria number 3).

**Response:** Thank you for the advice. We totally agree with reviewer 3 that statistical methods should be described in more detail.

According to the suggestion, the methods of *Statistical analysis* were modified as follows (page 6, line24–page 7, line 6): “All statistical analyses were performed using Review Manager (Revman, version 5.2) and Stata (version 12.0). If data from both intention-to-treat and per-protocol analyses were presented, the former were extracted and analyzed. Dichotomous data, including the rates of adequate bowel preparation, ADR or PDR, adverse events and diet compliance etc., were reported as odds ratio (OR) with 95% confidence interval (CI). Continuous data, including BBPS, OBPS, insertion and withdrawal time, were reported as standard mean difference (SMD) with 95%CI. Pooled estimates of OR or SMD were calculated using a random-effects model (M-H, heterogeneity), in which both within-study and between-study variations were considered (Control Clin Trials 1986;7:177-88.). Subgroup analysis were conducted according to the types of RE (communicable or not), evaluation tool (BBPS or OPBS), indication (screening or mixed) and preparation method (4L PEG, split-dose or low-volume laxatives). Statistical heterogeneity was accessed using the Cochrane Q test and by calculating the I^2^ value, with substantial heterogeneity defined as I^2^ greater than 50%, as described previously. A P value less than 0.05 was considered significant.”

We believe the current version of statistical methods should meet the publication criteria No.3 of *PLoS One*.

2, I consider also that the comments from the rest of the reviewers are important in order to improve the manuscript.

**Response:** Thank you for the suggestion. The constructive and suggestive comments from the reviewers and the editor are very helpful for improving our manuscript. The manuscript had been revised based on the suggestions. Point-to-point responses had been provided for all the questions and comments.

3, Flow Chart. From 760 manuscripts, 523 were excluded, and then 27 were assessed for eligibility, however, 760-523 are not 27. Please correct or explain it.

**Response:** Thank you for pointing out the mistake. We performed systemic literature search for several times. The outcomes of each time of searching may be mixed. We have corrected the flow chart.

4, Is the Universal preparation scale a validated scale? This is a limitation if not.

**Response:** Thank you for the comments. We agree that the use of invalidated UPAS scale to evaluate BP quality may bring some uncertainty to the results. The following contents were added into *Limitation* (page 14, line 17-19): “Fifthly, among the 5 scales used for the evaluation of BP quality, only BBPS (Gastrointest Endosc 2009;69:620-5; Gastrointest Endosc 2010;72:686-92;) and OBPS (Clin Gastroenterol Hepatol 2016;14:429-435 e3; Gastrointest Endosc 2004;59:482-6) were validated for the inter- and intra- observer consistence. Uncertainty of the results may exist with the uses of other 3 invalidated scales.”

5, Results (primary and secondary outcomes). Please include the confidence intervals of the proportions (not only of the OR)

**Response:** Thank you for the suggestion. We have added confidence intervals to all results.

6, Please English language needs some improvement. Make sure that the manuscript is edited.

**Response:** Thank you for the suggestion. The manuscript had been thoroughly reviewed and modified by a native English editor. The grammatical errors and typing errors had been carefully checked and corrected.

7, In page 11, the paragraph “and the conclusion ….until (38.2% vs. 38.4%)" needs to be rephrased

**Response:** Thank you for the question. There was no statistical difference in polyp detection rate (PDR) between patients receiving enhanced instructions and regular instructions (448/1245, 36.0% vs 363/1179, 30.8%; OR, 1.25; 95% CI, 0.93-1.68; P=0.140) either in Guo et al’s (Gastrointest Endosc 2017;85:90-97) meta-analysis or in Chang et al’s meta-analysis (Endosc Int Open 2015;3:E646-52) (RR, 1.14; 95% CI, 0.87-1.51). Likewise, Calderwood et al’ s study also demonstrated no statistical difference between intervention and control groups (182/477, 38.2% vs. 189/492, 38.4%; P=0.930). However, this updated meta-analysis showed opposite conclusion that patients receiving reinforced education had a higher PDR (637/2019, 33.2% vs. 483/1778, 28.2%; OR 1.24; 95%CI: 1.02-1.50; P=0.030) compared with those receiving regular educations. It may because this meta-analysis had enrolled more studies and patients.

**Reviewer #1:**
**Major**
1. Split dose. I miss an analysis of studies grouped by the split-dose with any laxative. Split-dosing is the variable that influences most the quality of bowel cleansing and it has been showed that it is not dependent of the laxative used. Is it possible to group the studies regarding just the split-dose with any laxative?

**Response:** Thank you for the question. We agree with the opinion of the reviewer that split-dose is an important factor associated with the quality of bowel preparation. It deserves further investigation with split-dose with any laxatives as one subgroup. We added the contents into *Results* as follows (page 9, line 6-10): “Split-dose with any laxatives. Among the included 18 studies, 12 used split-dose strategy. The laxatives included 3L or 4L PEG (n=8), 2L PEG+Asc (n=4) and SPMC (n=2). Patients with administration of split-dose laxatives in RE group showed better quality of bowel preparation compared with SE group (1810/2013, 90.0% vs. 1384/1814, 76.3%; OR 2.92; 95%CI: 2.31-3.68; P<0.001).”

The results of the subgroup analysis were supplemented as supplementary table 3, which was provided as follows.

Supplementary table 3. Bowel preparation quality in patients receiving split-dose with any laxatives.

|  | Studies | Adequate rate of bowel preparation | | | | BBPS score | | | |
| --- | --- | --- | --- | --- | --- | --- | --- | --- | --- |
|  |  | RE | SE | 95%CI | P value | Mean score | SMD | 95%CI | P value |
| Split-dose with any laxatives | 12 | 90.0% | 76.3% | 2.31-3.68 | <0.001 | 6.71 vs. 6.23 | 1.81 | 0.50-3.12 | 0.007 |
| 3L or 4L PEG | 8 | 88.2% | 71.7% | 2.40-4.06 | <0.001 | 6.95 vs. 6.07 | 0.69 | 0.13-2.09 | 0.020 |
| 2L PEG+Asc | 4 | 94.9% | 83.7% | 2.08-8.77 | <0.001 | 7.23 vs. 6.39 | 3.38 | 0.15-6.61 | 0.040 |
| SPMC | 2 | 91.3% | 75.0% | 0.51-24.66 | 0.200 | / | / | / | / |

Abbreviations: PEG, polyethylene glycol; RE, reinforced education; SE, standard education; SMD, standard mean difference; CI, confidence interval; Asc, ascorbic acid; SPMC, sodium picosulfate with magnesium citrate

2. I miss an exploration of the population included. There are some studies that include a population with no risk factors of poor bowel preparation (e.g. young, with no comorbidities or medications that may impair the bowel peristalsis) or a population “easy to educate” (e.g. young, high education, technologically competent). Is it possible to group the studies regarding the population included? It will allow to compare the studies and to look if the conclusions of those studies may be generalized to the general population. If it is not possible to analyze the population included I will like to include that as a limitation.

**Response:** Thank you for the suggestion. There are several patients-related parameters which had been identified as risk factors associated with higher quality of bowel preparation in the past decade. As the reviewer indicated, patients with younger age, without comorbidities (especially constipation, diabetes, Parkinson disease and spine injury) or medications (especially TCA and possibly CCB) and those with higher education level may represent an “easy-to prepare” group. It is interesting to investigate whether these patients may achieve adequate bowel preparation even without RE. Unfortunately, among the 18 studies focusing on investigating the effects of education on BP quality, none reported the results of bowel preparation in low-risk or high-risk patients.

We agree that it is a limitation since it is currently not possible to evaluate the effects of RE on BP quality in the population with low risks. The following contents were added into the *Limitations* (page 14 line 9-17): “Fourthly, patients with younger age, without comorbidities (especially constipation, diabetes, Parkinson disease and spine injury) or medications (especially tricyclic antidepressant (TCA) and possibly calcium channel blockers (CCB) and those with higher education level represent an relatively “easy-to-prepare” group (Am J Gastroenterol 2018;113:601-610). It is interesting to investigate whether these patients may achieve adequate bowel preparation even without RE. Unfortunately, among the 18 studies focusing on investigating the effects of RE on BP quality, none reported the results of bowel preparation in low-risk patients. Further studies are needed to investigate the effects RE on BP quality in the “easy to-prepare” population.”

3. The quality of the standard education varies in the different studies. It is possible that if the quality of the SE is low, such as written instructions only, the room for improvement may be greater than if the quality of the SE is better. Is it possible to group the studies in relation with the quality of the SE?
If it is not possible I will appreciate a comment in the limitations section.

**Response:** Thank you for the comments. We agree that the methods of SE may influence the quality of bowel preparation and the effects of RE. According to the suggestion, subgroup analysis were conducted and added into *Results* as follows (page 10, line 1-7): “SE methods. The methods of SE were described in detail in 16 studies, including 6 with written instructions alone, 1 with oral instructions alone and 9 using written plus oral instructions (Table 1). RE significantly improved the adequate rate in patients receiving only written instructions in the control group (1168/1308, 89.3% vs. 1005/1238, 81.2%; OR 2.29; 95%CI: 1.43-3.68; P<0.001). Similar results were found in patients receiving written plus oral instructions as the SE method (1300/1526, 85.2% vs. 963/1408, 68.4%; OR 2.77; 95%CI: 2.05-3.75; P<0.001).”

The methods of SE were illustrated in detail and supplemented in *Table 1* as follows.

Table 1. Methods of RE and SE among different studies.

| Study | Method of RE | Method of SE | Adequate BP rate |
| --- | --- | --- | --- |
| Back, 2018 | Audio-visual through smart phone | Oral + written | 96.4% vs. 73.6% |
| Calderwood, 2011 | Visual aid | Written | 90.6% vs. 89.0% |
| Elvas, 2016 | Additional personalized instruction | Oral + written | 62.1% vs. 35.4% |
| Ergen, 2016 | Booklet | NR | 62.0% vs. 35.0% |
| Kang, 2015 | Social media app | Oral + written | 90.0% vs. 75.6% |
| Lee, 2015 | Telephone & SMS | Oral + written | 96.0% vs. 86.1% |
| Liu, 2018 | Video plus retelling | NR | 90.0% vs. 75.1% |
| Liu, 2014 | Telephone | Oral + written | 81.6% vs. 70.3% |
| Lorenzo, 2015 | Smart phone app | Written | 100% vs. 96.1% |
| Modi, 2009 | Additional explanation | Oral + written | 69.0% vs. 57.5% |
| Park, 2015 | SMS | Written | 79.4% vs. 57.8% |
| Park, 2016 | Video | Written | 91.7% vs. 78.6% |
| Rice, 2016 | Online video | Oral + written | 73.8% vs. 68.0% |
| Sharara, 2017 | Smart phone app | Written | 82.5% vs. 77.5% |
| Spiegel, 2011 | New designed booklet | Oral + written | 75.8% vs. 46.3% |
| Tae, 2012 | Cartoon visual aids | Oral + written | 93.1% vs. 81.6% |
| Walter, 2019 | SMS | Oral | 90.7% vs. 80.6% |
| Wang, 2019 | WeChat & SMS | Written | 87.5% vs. 65.4% |

Abbreviations: RE, reinforced education; SE, standard education; BP, bowel preparation; SMS, short message service; NR, not reported

**Minor**
1. Originality. As the authors have stated there have been 4 previous meta-analysis exploring the association between an enhanced instruction for bowel prep and the bowel cleansing adequacy. Nevertheless the present study is the most updated analysis and it includes several RCTs in the last 4 years.

**Response:** Thank you for the comments. As the reviewer mentioned, compared with previous similar meta-analysis studies, the current study included another 4 latest RCTs and more patients (6536 vs. 2660-3795). The differences were described in detail in *Introduction* as follows (page 4, line 22-page5, line 2): “Four previous meta-analysis have been published to systemically compare the adequacy of BP among patients receiving enhanced instructions and standard education. The design and search strategies of these studies were different. Chang et al’s study was the first meta-analysis determining the effect of educational intervention on BP quality. Although 9 RCTs (n=2885) were included, 3 of them were abstracts. Desai et al enrolled 6 studies, which only detected smartphone applications on BP quality compared with standard education (n=810). Kurlander et al enrolled 7 studies with full articles (n=2660), however, 2 of them were not RCTs. In 2017, our team made comparisons of BP quality between patients receiving enhanced instructions plus regular instructions and regular instructions alone. 8 RCTs (n=3795) with full texts were enrolled. However, the literature search time were up to 2015. In the past 4 years, additional 10 high-quality clinical trials have been published. The results, however, seemed to be conflicting.”

2. There is a discrepancy between the 2 abstracts, in the first, line 8, it is stated that the literature search was done through March 2019. In the manuscript abstract, page 3, line 9, it says that the search was conducted until April 2019. Please correct it.

**Response:** Thank you for pointing out the mistake. The correct time is April 2019. The corresponding mistake has been corrected.

4. Page 6, lines 1,2 “diet restriction” is duplicated.

**Response:** Thank you for the suggestion. The duplicated “diet restriction” (pag6, line 1-2) has been deleted.

5. The effect of a reinforced education may be greater when the interval between the SE and the bowel preparation or the colonoscopy is longer, especially when the RE is made with a communicable tool. Is it possible to group the studies or the individual data to assess that variable?

**Response:** Thank you for the comments. We agree that RE may be more effective in patients with longer interval time from appointment day to the day of colonoscopy. Ten studies reported the details of interval time. Among them, 7 studies had shorter mean interval time (≤2 weeks) and 3 had longer (>2 weeks). Subgroup analysis showed that RE improved bowel preparation quality in both patients with longer (90.4% vs. 74.4%) or shorter (86.9% vs. 70.5%) mean interval time. The effects of RE seemed comparable between the two groups of patients (OR (95%CI): 2.79 (2.24-3.47) vs. 3.26 (1.31-8.14)).

Table. BP quality in patients with different interval time from appointment to colonoscopy.

| Interval time from appointment to colonoscopy | Study | Adequate rate of bowel preparation | | OR (95%CI) | P value |
| --- | --- | --- | --- | --- | --- |
|  |  | RE | SE |  |  |
| ≤2 weeks | 7 | 86.9% (1365/1570) | 70.5% (929/1317) | 2.79 (2.24-3.47) | <0.001 |
| >2 weeks | 3 | 90.4% (483/534) | 74.4% (406/546) | 3.26 (1.31-8.14) | 0.010 |

Table. Details of interval time from appointment to colonoscopy among studies.

| Study | Interval time from appointment to colonoscopy | | |
| --- | --- | --- | --- |
|  | Mean (days) | RE | SE |
| Back, 2018 | 17.3 | NR | NR |
| Calderwood, 2011 | NR | NR | NR |
| Elvas, 2016 | NR | NR | NR |
| Ergen, 2016 | NR | NR | NR |
| Kang, 2015 | 14.6 | 14.6 | 14.5 |
| Lee, 2015 | 5.4 | 5.4 | 5.4 |
| Liu, 2018 | 3.3 | 3.4±0.2 | 3.3±0.8 |
| Liu, 2014 | 3.4 | 3.4±0.8 | 3.5±0.9 |
| Lorenzo, 2015 | NR | NR | NR |
| Modi, 2009 | NR | NR | NR |
| Park, 2015 | 6.02 | 5.02±2.02 | 7.03±1.38 |
| Park, 2016 | NR | NR | NR |
| Rice, 2016 | 30 | NR | NR |
| Sharara, 2017 | NR | NR | NR |
| Spiegel, 2011 | 7 | NR | NR |
| Tae, 2012 | NR | NR | NR |
| Walter, 2019 | 4 | 4 | 4 |
| Wang, 2019 | 2 | 2 | 2 |

**Reviewer #2:**

**MAJOR**
1) The authors need to improve the language in the manuscript. There are spelling mistakes (pay attention to the capital letters in the names, et al needs a dot after the word, like that “et al.”), and some difficult to understand sentences. For example:
i. Page 6, line 2 withdraw. You should change it for withdrawal.
ii. Page 6, line 6. You should change it for rate. I also suggest to add in this sentence, “The pooled rate of adequate bowel cleansing was …”

**Response:** Thank you for the suggestion. The errors mentioned by the reviewer had been corrected. In addition, a native English editor had been invited to review and modify the manuscript thoroughly. All grammatical and typing errors had been carefully checked and corrected.

2) The figures and tables legend is not complete. Moreover, the references of the figures and supplementary figures don´t match in the text.

**Response:** We are sorry for the mistakes. The figures and tables legends were supplemented. Two authors had carefully and comprehensively double-checked the reference numbers in the manuscript, all figures and tables.

3) The authors have missed some articles in this meta-analysis, which fulfill the inclusion criteria. Do you have any reason to exclude them?
a. Galvez M, Zarate AM, Espino H, Higuera-de la Tijera F, Awad RA, Camacho S. A short telephone-call reminder improves bowel preparation, quality indicators and patient satisfaction with first colonoscopy. Endosc Int Open. 2017;5. E1172-E8.
b. Elvas L, Brito D, Areia M, Carvalho R, Alves S, Saraiva S, et al. Impact of Personalised Patient Education on Bowel Preparation for Colonoscopy: Prospective Randomised Controlled Trial. GE Port J Gastroenterol. 2017;24:22---30.
c. Prakash SR, Verma S, McGowan J, Smith BE, Shroff A, Gibson GH, et al. Improving the quality of colonoscopy bowel preparation using an educational video. Can J Gastroenterol. 2013;27:696---700.
**Response:** Thank you for telling us the important information. With careful evaluation, we agree that these three papers were relevant to reinforced education of bowel preparation. However, the primary outcome of this study was the rate of adequate bowel preparation. Only BBPS or OBPS score was reported for the evaluation of bowel preparation in the studies of Galvez et al. (Endosc Int Open. 2017;5. E1172-E8) and Prakash et al. (Can J Gastroenterol. 2013;27:696-700). From the original data of BBPS or OBPS score, the rates of adequate bowel preparation in could not be calculated. After full discussion, we decided not to include these two studies (Endosc Int Open. 2017;5. E1172-E8; Can J Gastroenterol. 2013;27:696-700) in this meta-analysis.

Then with further enrollment of Elvas’ study (GE Port J Gastroenterol. 2017;24:22-30), totally 18 studies with 6536 patients were included for this meta-analysis. Data from all of the 18 studies were checked and re-analyzed carefully. All figures and tables were refreshed accordingly.

**MINOR**
4) It could help to the reader to explain the definition of standard instructions in the study selection.

**Response:** Thank you for the advice. Standard instructions mean oral instructions, written instructions or oral plus written instructions provided by physicians or nurses before colonoscopy. The definition was supplemented in *Methods* of the manuscript as follows (page 5, line 25-30): “SE meant oral instructions, written instructions or oral plus written instructions associated with bowel preparation, which was provided by physicians or nurses before colonoscopy. The contents of SE included diet restriction, the time and methods of drinking purgatives. RE referred to additional, enhanced instructions based on SE, which was realized by providing some certain of methods or tools. The contents of SE and RE were generally the same.”

5) Pag 7, paragraph 1. It could be better to add the reference after each bowel preparation scale to let the reader know which are the studies are you referring to.

**Response:** Thank you for this suggestion. Corresponding references have been added into *Methods* (page 6, line 1-4) as suggested. Here are the 5 original references related to BP scales.

1) **BBPS**: Lai EJ, Calderwood AH, Doros G, et al. The Boston bowel preparation scale: a valid and reliable instrument for colonoscopy-oriented research. Gastrointest Endosc 2009;69:620-5
2) **OBPS**: Rostom A, Jolicoeur E. Validation of a new scale for the assessment of bowel preparation quality. Gastrointest Endosc 2004;59:482-6.
3) **UPAS**: Modi C, Depasquale JR, Digiacomo WS, et al. Impact of patient education on quality of bowel preparation in outpatient colonoscopies. Qual Prim Care. 2009;17(6):397–404.
4) **HCS**: Halphen M, Heresbach D, Gruss HJ, et al. Validation of the Harefield Cleansing Scale: a tool for the evaluation of bowel cleansing quality in both research and clinical practice. Gastrointest Endosc 2013;78:121-31.
5) **Aronchick scale**: Aronchick CA, Lipshutz WH, Wright SH, et al. A novel tableted purgative for colonoscopic preparation: efficacy and safety comparisons with Colyte and Fleet Phospho-Soda. Gastrointest Endosc 2000;52:346-52.

6) Pag 7, communicable and non-communicable tools. I suggest to add the definition and the reference to the correct supplementary table.

**Response:** Thank you for the suggestion. Communicable tools refer to the RE methods which allow patients to communicate with physicians or nurses if they had any questions about bowel preparation during the procedure of instruction or met problems during the preparation. Communicable tools in this study included social media application (WeChat) (Kang, 2015; Wang, 2019) and telephone call (Liu, 2014; Lee, 2015). According to the suggestion of the reviewer, the definition and corresponding references were added in Table 1 (page 16, line 2-18).

The following sentence was added into *Results* (page 8, line 11-20): “4 studies used communicable tools, including social media application (WeChat) (Kang, 2016; Wang, 2019) and telephone call (Liu, 2014; Lee, 2015). Through the communicable tools, patients could communicate with physicians or nurses if they had any questions about bowel preparation during the procedure of instruction or met problems during the preparation. By using communicable tools for RE, patients achieved better BP quality (1035/1166, 88.8% vs. 678/914, 74.2%; OR 2.84; 95%CI:1.97-4.11; P<0.001). In other 14 studies with non-communication tools as the RE methods, patients also showed a higher rate of adequate BP compared with the control group (1902/2198, 86.5% vs. 1681/2254, 74.6%; OR 2.52; 95%CI:1.92-3.30; P<0.001).”

7) Pag 8- Did you analyze the BP quality by using other purgatives as low volume preparations?

**Response:** Thank you for the question. Several types of low-volume preparation are becoming popular in clinical practice due to its similar efficacy and lower adverse events compared with 4L PEG (Clin Gastroenterol Hepatol. 2019;S1542-3565(19)31246-7). It is interesting to investigate whether RE is useful for the improvement of BP quality in patients undergoing low-volume preparation.

Low-volume preparations were reported in 6 enrolled studies. The laxatives for preparation included 2L PEG+Asc (n=3), 2L PEG (n=3) and NaP or magnesium citrate (n=2). Subgroup analysis was conducted to evaluate the influences of RE on BP quality in the population undergoing low-volume preparations.

The data was added into *Results* as follows (page 9, line 10-17): “Low-volume laxatives. Several types of low-volume preparations have recently been shown with similar efficacy and lower adverse events compared with 4L PEG (Endoscopy 2019; 51: 775–94; Clin Gastroenterol Hepatol. 2019;S1542-3565(19)31246-7). Here 6 studies with 2492 patients used low-volume preparations, including 2L PEG+Asc (n=3), 2L PEG or NaP or magnesium citrate (n=3). Compared with SE, RE showed higher rate of adequate BP in patients undergoing 2L PEG+Asc (576/609, 94.6% vs. 463/536, 86.4%; OR 2.84; 95%: 1.83-4.40; P<0.001) or 2L PEG (564/676, 83.4% vs. 451/671, 67.2%; OR 2.63; 95%CI: 1.75-3.97; P<0.001).”

8) It could be better to add in the results section some data regarding the diminutive adenoma detection rate as you discussed it latter.

**Response:** Thank you for the suggestion. The data of diminutive (1-5mm) adenoma detection rate were described in only one study. As the reviewer suggested, the following contents were added into *Results* (page 10, line 18-20): “Diminutive adenoma detection rate was reported in 1 study (Kang, 2016), which was also higher in the RE group (51/387, 13.2% vs. 30/383, 7.8%, P=0.019).”

**Reviewer #3:**

Thank you for the opportunity to review this paper. This is an interesting manuscript presenting a results of a meta-analysis of randomized controlled trials on reinforced education improves the quality of bowel preparation for colonoscopy.
My review mainly concerns only the statistical aspects of the study. Some questions reported below were raised and in my view, it is not acceptable in this version for the publication in this journal.

Comments
1. Statistical analysis paragraph is a duplicate of the paragraph of the precedent paper [Guo X, Yang Z, Zhao L, et al. Enhanced instructions improve the quality of bowel preparation for colonoscopy: a meta-analysis of randomized controlled trials. Gastrointest Endosc 2017;85:90-97 e6.]. More detail are necessary. The meta-analysis was conduct on OR and on mean differences in the statistical analysis paragraph should be detailed.

**Response:** Thank you for the important comments. We totally agree with the reviewer that statistical methods should be described in more detail.

According to the suggestion of the reviewer, the methods of *Statistical analysis* were modified as follows (page 6, line 24-page7, line 6): “All statistical analyses were performed using Review Manager (Revman, version 5.2) and Stata (version 12.0). If data from both intention-to-treat and per-protocol analyses were presented, the former were extracted and analyzed. Dichotomous data, including the rates of adequate bowel preparation, ADR or PDR, adverse events and diet compliance etc., were reported as odds ratio (OR) with 95% confidence interval (CI). Continuous data, including BBPS, OBPS, insertion and withdrawal time, were reported as standard mean difference (SMD) with 95%CI. Pooled estimates of OR or SMD were calculated using a random-effects model (M-H, heterogeneity), in which both within-study and between-study variations were considered (Control Clin Trials 1986;7:177-88.). Subgroup analysis were conducted according to the types of RE (communicable or not), evaluation tool (BBPS or OPBS), indication (screening or mixed) and preparation method (4L PEG, split-dose or low-volume laxatives). Statistical heterogeneity was accessed using the Cochrane Q test and by calculating the I^2^ value, with substantial heterogeneity defined as I^2^ greater than 50%, as described previously. A P value less than 0.05 was considered significant.”

2. Why in Figure 2 the OR estimated for Lee,2015 is different to the estimate of the previous paper? (3.91 vs 4.38)

**Response:** Thank you for the question. In the previous meta-analysis (Gastrointest Endosc 2017;85:90-97), the data of intervention group and control group were respectively 243/251 and 118/135 (OR, 4.38; 95%CI, 1.84-10.43). The data were retrieved from PP (per-protocol) analysis. In this study, the data were 243/253 and 118/137 (OR, 3.91; 95%CI, 1.76-8.68), which were retrieved from ITT (intention-to-treat) analysis. We believe ITT analysis might be better for the evaluation of the primary outcome. The results of ITT analysis were presented for all included studies in this study, which conferred on the differences of the results.

The following sentence was added into *Methods* (page 6, line 26-27): “If data from both intention-to-treat and per-protocol analyses were presented, the former were extracted and analyzed.”

3. There are many subgroup analysis. The capabilites of subgroup analyses to detect meaningful differences between studies is often limited. Subgroup analyses also need sufficient power.
**Response:** Thanks for the comments. We admitted that it is one of limitations of this study. The following sentences were added into *Limitations* (page 14, line 19-24): “Last but not the least, to evaluate the effects of RE in different conditions, several subgroup analyses were performed. Although significant differences were found in most of the analyses (P<0.001). The power of the subgroup analyses may be not sufficient. The capabilities of subgroup analyses to detect meaningful differences between studies is often limited, thus it should be caution to explain the results.”

**Minor comments**
1, There are many error of typing. Please, verify the manuscript for English and typos;

**Response:** Thank you for the suggestion. The manuscript had been thoroughly reviewed and modified by a native English editor. The grammatical errors and typing errors had been carefully checked and corrected.

2, p-value should be reported with three decimal number and with exact value; uniform the notation or P or p.

**Response:** Thank you for the suggestion. All P value were modified as with three decimal number or with exact value. “P”s were used as the uniform spelling.

3, In the funnel plot the line of the 95% CI can make easier to read the figure

**Response:** Thank you for the advice. The figure of funnel plot with both OR and 95%CI lines was re-produced by using Stata software. We agree that new figure was easier to read.

Additionally, the following sentence was added into *Results* (page 11, line 18-19): “Publication Bias. Begg’s test was conducted by Stata and the funnel plot showed no significant publication bias was found (P=0.950) (Supplementary figure 3).”
