# Supplementary material for: Reinforced education improves the quality of bowel preparation for colonoscopy: An updated meta-analysis of randomized controlled trials
Source: PLoS One. 2020 Apr 28;15(4):e0231888. doi: 10.1371/journal.pone.0231888 (PMC7188205; doi:10.1371/journal.pone.0231888)
Supplement: Attachment — Submitted filename: response to reviewer.docx [file pone.0231888.s022.docx]

Dear editor,

We thank the editor and the reviewer for reviewing our Manuscript ID PONE-D-19-27355R1 entitled “Reinforced education improves the quality of bowel preparation for colonoscopy: an updated meta-analysis of randomized controlled trials”. We have revised the manuscript according to the constructive suggestions. A point-by-point response is included below.

In response to the reviewers' comments, additional information has been added to the revised manuscript. These are marked by yellow highlights in the version with tracked changes. The version with all changes accepted (clean version) are also provided. Deleted text no longer appears in the final clean version.

Major

1) Although for this journal novelty is irrelevant, the authors state that this is an updated meta-analysis in the abstract but they don't tell us why this is needed, what were the findings in the last one, and how many new studies have been added.

**Response:** Thank you for the questions and suggestions.

There are some reasons why we carried out this updated meta-analysis. Firstly, there existed a number of new qualified studies related to reinforced education (ER) for bowel preparation (BP), which should be reviewed and analyzed. Secondly, with the development of science and techniques, tools and methods REs in recent years varied from the past, especially for the wide use of smartphones and their applications. Thirdly, some secondary outcomes enrolled a limited number of qualified studies and patients’ number, which may influence the accuracy and reliability of outcomes. Therefore, we decided to write an updated systemic review and meta-analysis.

Compared to the previous meta-analysis conducted by our team (Gastrointest Endosc 2017;85:90-97 e6)*,* this updated-meta analysis conducted the same conclusion for the primary outcome: BP quality. However, there were some new findings for secondary outcomes: 1) reinforced education (RE) improved both adenoma detection rate (ADR) (OR 1.35; 95%CI: 1.06-1.72; P=0.020) and polyp detection rate (PDR) (OR 1.24, 95%CI: 1.02-1.50; P=0.030), while it was negative in PDR (OR 1.25; 95% CI, 0.93-1.68; P=0.140) and not reported in ADR in the previous study; 2) patients in the RE group had a shorter insertion time (MD -0.76; 95%CI: -1.48-(-0.04); P=0.040), while there was no statistical difference in the previous study (MD, -0.57; 95% CI, -1.38 to 0.24; P=0.170); 3) less nausea/vomiting (OR 0.78; 95%CI: 0.64-0.97; P=0.020) and abdominal distension (OR 0.72; 95%CI: 0.68-0.92; P=0.020) were achieved in the RE group, while there was no statistical difference in the previous study for nausea/vomiting (OR 0.77; 95% CI, 0.60-0.99; P=0.050) and abdominal distention (OR 0.86; 95% CI, 0.66-1.12; P=0.260). The following sentences were added in *Discussion* section (line 3, page 12):“Although the primary outcome was similar to the four previous systemic review and meta-analyses, this updated meta-analysis conducted some new conclusions in secondary outcomes: 1) RE improved both ADR and PDR, which firstly demonstrated that patients receiving RE had a higher PDR in the form of systemic review and meta-analysis; 2) patients in the RE group had a shorter insertion time; 3) less nausea/vomiting and abdominal distension were achieved in the RE group.”

In the *Introduction* section, we have described that:“In 2017, our team made comparisons of BP quality between patients receiving enhanced instructions plus regular instructions and regular instructions alone. 8 RCTs (n=3795) with full texts were enrolled. However, the literature search time were up to 2015. In the past 4 years, additional 10 high-quality clinical trials have been published^14, 16, 19-21, 24, 29-32^.”

2) The language is quite poor, difficult to understand and not very academic. Consider the following passage: "For the primary endpoint, sensitivity analysis showed that a significantly better heterogeneity was noted (Heterogeneity: I2 from 56% to 34%) with removal of Calderwood’s study12. The following two reasons were considered: (1) it had the biggest sample size but resulted in a negative conclusion; (2) factors that caused the negative conclusion due to not providing patients with specific information of enhanced education, such as the solution of purgative use or diet restrictions." There is no better heterogeneity. the authors mean lower. also it is unclear what a negative conclusion is.

**Response:** Thank you for the question and advice. Firstly, we apologize that the data of 56% and 34% were not correct, because they were the first edition’s data which included 17 studies and we forgot to correct. As we know, I^2^ from 25% to 50% was mild heterogeneity, I2 from 50% to 75% was moderate heterogeneity and >75% belongs to sever heterogeneity. In the first edition, only after extracting Calderwood’s study, I^2^ was 34%, while after extracting other studies one by one, all I^2^ values were >50%. Therefore, we detected the reasons of the decreasing of I^2^ from Calderwood’s study statistically and clinically. Among the all 18 included studies, 4 studies (Calderwood’s, Modi’s, Rice’s and Sharara’s study) conducted a negative conclusion that RE did not improve the quality of BP, while other 14 studies proved that RE could improve BP quality. And among all 18 included studies, Calderwood’s study had the biggest sample size (969/6536, 14.8%). So, it is worth detecting why this study drew a negative conclusion with the biggest sample size clinically. We found that the content of RE doctors provided to patients were not specific and quantified enough, e.g. the time when purgative was taken, the forbidden food and specific recommended food when undergoing BP.

In fact, the correct overall I^2^ of 18 included studies was 53%. Sensitivity analysis was conducted by extracting studies one by one. After extracting Calderwood’s study and Back’s study, the I^2^ changed to 30% and 44% separately, while after the extraction of other studies one by one, all I^2^ were >50%. The following sentences were added in *Method* (line 9, page 11)*:* “Sensitivity analysis For the primary endpoint, the I^2^ value of heterogeneity was 53%. Sensitivity analysis was conducted with the extraction of study one by one. It showed that after extracting Calderwood’s study and Back’s study, the I2 changed to 30% and 44% separately, while after the extraction of other studies one by one, all I2 values were >50%.”

3) No information on publication bias tests and assessment in the methods section, only mentioned in the results section. Publication bias tests and plots only relevant if you have >10 studies otherwise underpowered to detect much and tend to lead to conclusions that are not justified http://www.ncbi.nlm.nih.gov/pubmed/11106885. Here, with your number of studies, you can state as a considerable strength.

**Response:** Thank you for the suggestion. We have added publication bias tests and assessment in the *Method* section. The following sentence was added in *Method* section (line 4, page 7):“Publication bias was assessed by visual inspection of a funnel plot using Review Manager and was detected by Stata software”. The following sentence was added in *Result* section (line 9, page 11):“The funnel plots performed by Revman that was asymmetric (Supplementary figure 3). Begg’s test was conducted by Stata and the funnel plot showed no significant publication bias was found (P=0.950) (Supplementary figure 4).”

4) Report the confidence intervals for I^2 (calculated using heterogi or metaan in Stata) as argued in http://www.ncbi.nlm.nih.gov/pubmed/17974687. A simple formula exists in the seminal 2002 Higgins paper that proposed I^2.

**Response:** Thank you for the suggestion. We have carefully read the two passages you mentioned above, which made us had a deeper understanding of heterogeneity and its related values. Higgins’s study (Statist Med 2002;21:1539-1558) proposed three kinds of values to evaluate heterogeneity: H, R, and I^2. Although methods and formulas were introduced in the appendix of this study, we extraordinarily apologize that it was too difficult to calculate the CI of I^2. It seemed there was lack of formulas of 95%CI of I^2, which we finally failed to figure out. As stated in Ioannidis’s study (BMJ 2007;335(7626):914-6):“All statistical tests for heterogeneity are weak, including I^2. The clinical implications of this are considerable and must be examined on a case by case basis. Putting too much trust in homogeneity of effects may give a false sense of reassurance that one size fits all.

Minor
1) Some careful proof-reading is needed. Eg. the same sentence appears twice in the start of the methods section.

**Response:** Thank you for the suggestion and pointing out the mistake. The duplicated sentence about statistical analysis in the *Methods* section has been deleted. In addition, a native English editor had been invited to review and modify the manuscript thoroughly. All grammatical and typing errors had been carefully checked and corrected.

2) authors mean standardised rather than standard mean difference. Also are the relevant outcomes reported in different scales necessitating the use of SMDs? if you can avoid SMDs it is preferable since they are difficult to interpret, compared to MD, and a back-transformation may be needed to convery the effect to a scale the readers are familiar with.

**Response:** Thank you for the suggestion. We agree that using MD is more appropriate in this study. All continuous data have been changed to be reported as mean difference (MD) with 95%CI and the outcomes did not change. The sentence in *Abstract* section (line 14, page 3) have been changed as follows:” Continuous data were reported as mean difference (MD) with 95%CI”.

3) Spell out Mantel-Haenszel.

**Response:** Thank you for the suggestion. Because all outcomes were used RE models, which could use M-H method. The “M-H” in Method section has been deleted (line 31, Page 6).

4) Year may be worth considering in bias assessment, especially if you don't have enough studies for a formal test: http://www.ncbi.nlm.nih.gov/pubmed/25988604. With newer studies we would be more confident.

**Response:** Thank you for the suggestion. Among the included 18 RCTs, the earliest study was published in 2009 and most studies were reported in the last ten years. Publication years were summarized in *Table 1.*

Table 1. Time distribution of included studies

| Year | Studies |
| --- | --- |
| 2001-2005 | 0 |
| 2006-2010 | 1 |
| 2011-2015 | 8 |
| 2016-2019 | 9 |

5) How was the random-effect model implemented, i.e. how was heterogeneity estimated? There are numerous ways to do so. Did they use the standard DerSimonian-Laird method? If so, please state so. Also there are better performing methods, for example please see https://www.ncbi.nlm.nih.gov/pubmed/28815652 (or http://www.ncbi.nlm.nih.gov/pubmed/23922860) and the metaan command in Stata where these are implemented (<https://www.stata-journal.com/article.html?article=st0201>).

**Response:** Thank you for the suggestion. In this meta-analysis, all primary and secondary outcomes were analyzed by Revman software, which could not use DerSimonian-Laird (D-L) method that is mostly used in random-effect models. As a supplementary part, the primary outcome was analyzed by Stata software using D-L method. The outcome of OR and 95%CI were the same between the two software (2.59 (2.09-3.19)). The forest plot of the primary outcome using Stata have been added as Supplementary Figure 5.

6) Note that MH is traditionally a fixed effect approach and the random effects version in RevMan is an inverse variance-MH hybrid method, which has not been properly evaluated and is not avaialble elsewhere (except for metaan in Stata).

**Response:** Thank you for the reminder and suggestion. As a supplementary part, the primary outcome was analyzed by Stata software using D-L method. The outcome of OR and 95%CI were the same between the two software (2.59 (2.09-3.19)).

7) Cochran Q (i.e. chi-square) is notoriously underpowered to detect heterogeneity, especially for small meta-analyses http://www.ncbi.nlm.nih.gov/pubmed/9595615. I would not use

**Response:** Thank you for the advice. We agree that Cochran Q (i.e. chi-square) test is less statistically valid than I^2^. “using the Cochrane Q test” has been deleted in *Abstract* and *Methods* sections.

8) The authors do well to use RE models, since they outperform FE models in the presence of ANY heterogeneity. However, how did they assess heterogeneity? There are numerous ways to do so. Did they use the standard DerSimonian-Laird method? If so, please state so. Also there are better performing methods, for example please see http://www.ncbi.nlm.nih.gov/pubmed/23922860 and the metaan command in Stata where these are implemented.

**Response:** Thank you for the question and suggestion. In fact, in this meta-analysis, the I^2^ were both 53% using FE and RE models, belonging to moderate heterogeneity. Considering the method and tools of reinforced education varied from all included studies, which may lead to the heterogeneity, therefore, RE model was used. For heterogeneity test, firstly, subgroup analyses were made. Secondly, sensitivity analysis was conducted with the extraction of studies one by one, which finally did not show a significantly better heterogeneity. Thirdly, D-L method was used in Stata software, which conducted the same conclusion with using Revman software.
